# Transcription reinitiation by recycling RNA polymerase that diffuses on DNA after releasing terminated RNA

**DOI:** 10.1038/s41467-019-14200-3

**Published:** 2020-01-23

**Authors:** Wooyoung Kang, Kook Sun Ha, Heesoo Uhm, Kyuhyong Park, Ja Yil Lee, Sungchul Hohng, Changwon Kang

**Affiliations:** 1 Department of Physics and Astronomy, and Institute of Applied Physics, Seoul National University, Seoul, 08826 Republic of Korea; 2 Department of Biological Sciences, Korea Advanced Institute of Science and Technology, Daejeon, 34141 Republic of Korea; 3 School of Life Sciences, Ulsan National Institute of Science and Technology, Ulsan, 44919 Republic of Korea; 4 Present Address: Department of Life Science, University of Suwon, Gyeonggi-do, 18323 Republic of Korea; 5 Present Address: Department of Physics, University of Oxford, Oxford, OX1 3PU UK

**Keywords:** RNA, Single-molecule biophysics, Transcription

## Abstract

Despite extensive studies on transcription mechanisms, it is unknown how termination complexes are disassembled, especially in what order the essential components dissociate. Our single-molecule fluorescence study unveils that RNA transcript release precedes RNA polymerase (RNAP) dissociation from the DNA template much more often than their concurrent dissociations in intrinsic termination of bacterial transcription. As termination is defined by the release of product RNA from the transcription complex, the subsequent retention of RNAP on DNA constitutes a previously unidentified stage, termed here as recycling. During the recycling stage, post-terminational RNAPs one-dimensionally diffuse on DNA in downward and upward directions, and can initiate transcription again at the original and nearby promoters in the case of retaining a sigma factor. The efficiency of this event, termed here as reinitiation, increases with supplement of a sigma factor. In summary, after releasing RNA product at intrinsic termination, recycling RNAP diffuses on the DNA template for reinitiation most of the time.

## Abstract (web summary)

Bacterial transcription is terminated when RNA polymerases encounter terminator sequences. Using a single-molecule fluorescence assay, here the authors show that the release of transcript RNA precedes RNA polymerase dissociation and that the remaining RNA polymerase can reinitiate at nearby promoters.

## Introduction

Gene expression is regulated at not only initiation but also elongation and termination stages of transcription process for RNA biosynthesis, among other subsequent processes, such as RNA processing and transport, and protein synthesis, processing, and targeting.^1,2^ Transcription initiates at DNA promoters, and terminates with particular RNA sequences that are encoded by DNA terminators to effect particular pause of RNA polymerase (RNAP) and eventual disassembly of transcription complex to release RNA product.

*Escherichia coli* transcription initiates with RNAP holoenzyme, i.e., core enzyme plus a sigma factor, and the elongation process can be affected by transcription factors, such as NusA and NusG, among others. Termination can occur without any factors, although its termination efficiency can be affected by some factors, such as NusA. Such factor-independent (intrinsic) termination is caused by RNA structures with a GC-rich hairpin followed by a U-rich tract.^3–6^ The U-rich tract makes elongation complex pause to allow for GC-rich RNA hairpin formation that effects conformation changes to destabilize the complex.

Although the structural changes have not been characterized yet, all termination models^7,8^ include a structural change where DNA–RNA hybrid becomes unstable, which allows RNA to get locally separated from DNA and form hairpins. Single-molecule experiments using optical tweezers have shown termination mechanisms with RNAP forward hypertranslocation and RNA shearing,^9^ which had not been involved in an allosteric mechanism demonstrated by biochemical studies.^10^

However, it is still unknown yet how the complex is broken apart. Especially, which of the three essential components departs the transcription complex first, RNAP enzyme, RNA product, DNA template, or all together at once? In this study, we used single-molecule fluorescence measurements to primarily examine the dissociations of fluorescently labeled RNA and DNA from unlabeled RNAP, and their post-terminational fates in *E. coli* intrinsic termination. Our findings include that RNAP on an intrinsic terminator generally releases RNA transcript without departing from DNA template, and the remaining RNAP one-dimensionally diffuses on DNA in both directions and even reinitiates transcription at a nearby promoter.

## Results

### Construction of fluorescent transcription complex

**Fig. 1:**
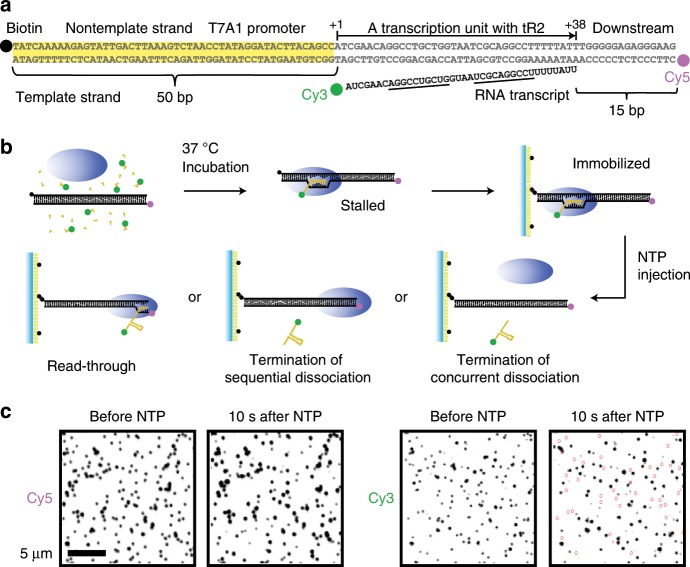
Intrinsic termination observed in single-molecule fluorescence experiments. **a** DNA template L+15 with T7A1 promoter and tR2 terminator was prelabeled with biotin (black dot) at the upstream end and with Cy5 (magenta dot) at the downstream end. RNA transcript was labeled with Cy3 (green dot) at the 5′-end. **b** Experimental scheme. Transcription complexes were stalled, immobilized, washed, and then resumed to follow readthrough or termination pathway. **c** Cy3-RNA and Cy5-DNA images (black spots) before and 10 s after NTP addition. Purple circles spot the termination complexes that have lost Cy3-RNA.

A primary DNA template (Fig. 1a) was synthesized to consist of a 50-bp upstream part, including the strong promoter A1 of bacteriophage T7, a 38-bp transcription unit with the intrinsic terminator tR2 of phage λ, and a 15-bp downstream part. It was prelabeled with Cy5 at the 5′-end of template strand (downstream end) and additionally with biotin at the 5′-end of nontemplate (coding) strand (upstream end).

In an experiment with radioactive UTP, the major termination site (TS) was +38 position relative to the transcription start site +1, and termination efficiency was 39%. This double-labeled template with a TS-downstream length of 15 bp is denoted by L+15. Likewise, other DNA templates are denoted by L+# with TS-downstream base-pair numbers.

A fluorescent transcription complex with Cy3-labeled RNA was prepared by incubating L+15 with Cy3-labeled ApU, ATP, CTP, GTP, and *E. coli* RNAP holoenzyme with σ^70^ for 30 min in a transcription buffer (Fig. 1b). Transcription is initiated preferentially with Cy3-ApU, which is incorporated into the +1 and +2 positions of RNA, but stalled with 12-nucleotide long RNA waiting for the missing UTP. The stalled complexes were immobilized on polymer-coated quartz slides using biotin–streptavidin conjugation and extensively washed with a buffer to remove all unimmobilized complexes and unbound components.

### Fluorescent detection of termination and readthrough

The synchronized, immobilized complexes were then subjected to resumption of elongation by adding all four ribonucleotides (NTPs), while fluorescence images of Cy3-RNA and Cy5-DNA in individual complexes were monitored using total internal reflection fluorescence microscopy. The resumed elongation is followed by either readthrough or termination at TS leading to continued or discontinued downstream transcription, respectively. In the fluorescence images (Fig. 1c), vanishing of Cy3-RNA images from the Cy5-DNA spots after NTP addition indicates release of product RNA from immobilized DNA, which defines termination of transcription.

**Fig. 2:**
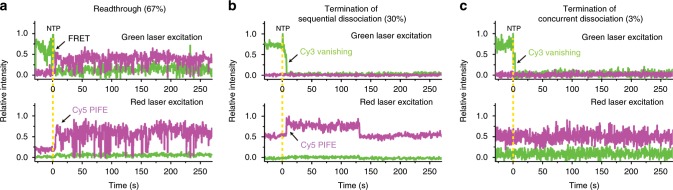
Representative fluorescence traces at green and red laser excitations. **a** In the readthrough events, RNAP reaches the downstream end of DNA without releasing RNA, showing both protein-induced fluorescence enhancement (PIFE) of Cy5 and fluorescence resonance energy transfer (FRET) from Cy3 to Cy5. **b**, **c** Among the remaining termination events, which show the release of Cy3-RNA, 91% retain RNAP on DNA showing Cy5 PIFE **b**, and 9% show RNAP dissociation without Cy5 PIFE **c**. Yellow vertical lines indicate NTP addition timing.

In readthrough events, the terminator is ignored by RNAP that passes through TS and continues to transcribe its downstream. With readthrough complexes, from which Cy3-RNA signal is not vanished, protein-induced fluorescence enhancement (PIFE) of Cy5 occurs with red laser excitation (Fig. 2a), indicating that RNAP reaches the downstream end of DNA and contacts Cy5 there.^11^ Additionally, fluorescence resonance energy transfer from Cy3 to Cy5 occurs with green laser excitation, indicating that the 5′-end (Cy3) of RNA also approaches the downstream end (Cy5) of DNA presumably due to cotranscriptional folding of transcript RNA.

In termination events, the terminator is recognized by RNAP that ends elongation at TS and does not transcribe its downstream. From termination complexes, Cy3-RNA signal vanishes 6.1 ± 0.9 s (mean ± s.d., *n* = 818 from five independent experiments) after NTP addition (Fig. 2b, c). The termination efficiency measured by the frequency of this pathway is 33 ± 4% (Supplementary Table 1), similar to the above measured using radioactive incorporation. When ITP is used instead of GTP to destabilize terminator hairpin RNA, this pathway is reduced to 10 ± 1%. Furthermore, this pathway is not observed with a terminator-lacking template L+15 M.

### RNAP remaining on DNA after releasing RNA

Surprisingly, Cy5 PIFE is observed in most (91 ± 5%) of the termination complexes, which lose Cy3-RNA signal (Fig. 2b), while the other termination complexes exhibit only Cy3 vanishing without Cy5 PIFE (Fig. 2c). In the majority, the Cy5 PIFE starts almost concurrently with Cy3 vanishing and is maintained for 536 ± 82 s (Supplementary Fig. 1), while Cy5 photobleaching takes a much longer time of 2350 s (Supplementary Fig. 2). Accordingly, after intrinsic termination, most RNAPs keep contacting DNA, as its possibility was previously speculated.^12^

This post-terminational retention of RNAP is little affected by addition of transcription factors NusA, NusG, or both, as the post-terminational PIFE occurrence is 86 ± 6% with NusA, 90 ± 11% with NusG, and 87 ± 3% with both NusA and NusG (Supplementary Table 2). Interestingly, NusA raises the termination efficiency much to 58 ± 10% without much affecting the termination timing (7.9 ± 0.2 s, since NTP injection), while NusG little affects the efficiency (35 ± 3%) or timing (4.1 ± 0.2 s). The presence of both factors (58 ± 4%, 7.0 ± 0.8 s) yields the property with NusA only. Any other RNAP-associating factors possibly contaminating the used RNAP preparation appear to little affect the RNAP retention, because similar PIFE occurrence (95 ± 6%) is observed with an extensively lab-purified cloned RNAP exhibiting similar termination properties (38 ± 7%, 4.7 ± 0.4 s).

When the terminator is further distanced from the promoter with the transcription unit enlarged from 38 bp of L+15 to 257 bp in a longer template T257/L+15, the PIFE occurrence remains similar (86 ± 2%), suggesting that the RNAP retention is not limited to promoter-proximal terminator. The retention is additionally observed with other intrinsic terminators, such as *E. coli his* operon attenuator (87% PIFE with termination efficiency of 84%) and phage φ82 t500 terminator (70% PIFE with termination efficiency of 68%), suggesting that the post-terminational retention of RNAP on DNA is general.

**Fig. 3:**
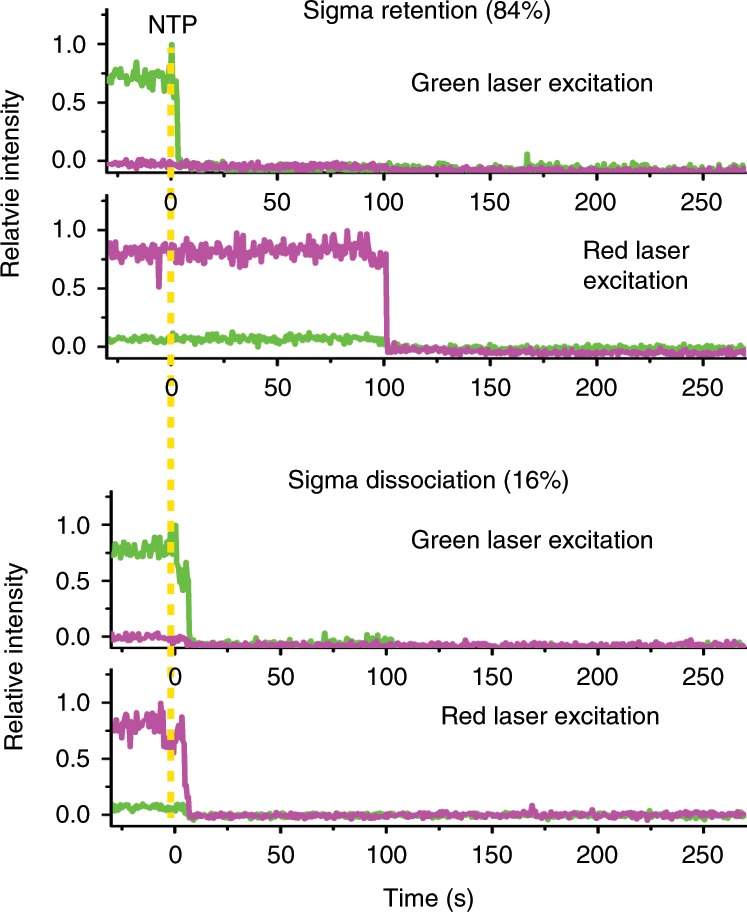
Retention of σ^70^ factor. An L+15 derivative complex with Cy5 (magenta dot) placed on σ^70^ factor (light grey oval) and with Cy3 (green dot) labeled on the 5′-end of transcript RNA (dark grey line) is immobilized through biotin (black dot). Retention of Cy5-σ on DNA after termination (upper trace) occurs more frequently than its dissociation from DNA (lower trace).

To gain further support, we repeated the experiments with Cy5 placed on σ factor rather than DNA (Fig. 3), because the initiation factor has been recently reported to remain in some elongation complexes.^13–15^ An active derivative of *E. coli* σ^70^ was labeled with Cy5 at Cys-366.^16^ Cy5-σ is retained in 75% of the initially stalled complexes (Supplementary Fig. 3), which falls within a previously reported 70–90% range of σ retention during elongation.^15^

This Cy5-σ of holoenzyme mostly (84%) remains on immobilized DNA even after Cy3-RNA vanishing of termination (Fig. 3), i.e., the σ^70^ retained in the initially stalled complex mostly remains on DNA after RNA release. In no cases, Cy5-σ vanishes before Cy3-RNA. The dissociation time of σ^70^ (588 ± 85 s, Supplementary Fig. 4) is similar to that of RNAP (536 ± 82 s, Supplementary Fig. 1), suggesting that the holoenzyme maintains a stable complex throughout its retention on DNA.

### One-dimensional diffusion of RNA-free RNAP on DNA

**Fig. 4:**
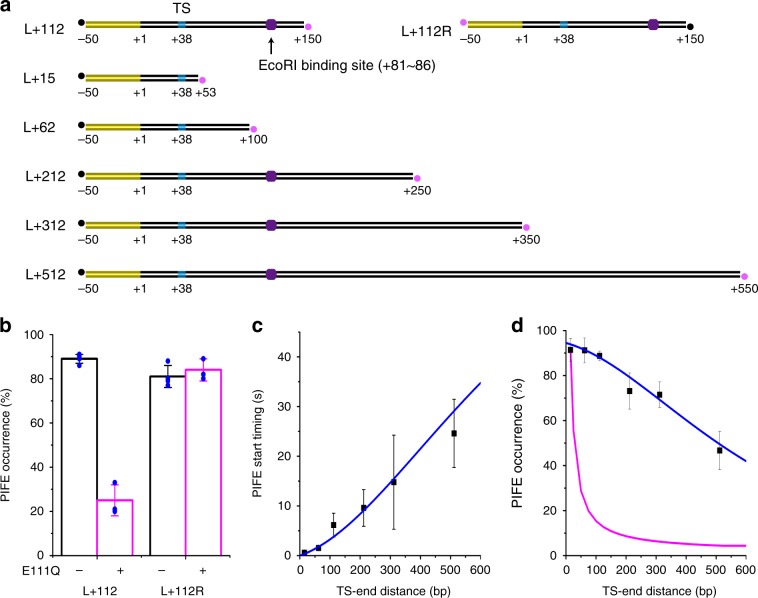
Diffusion of RNAP on DNA after termination. **a** DNA templates with varying distance between TS (cyan oval) and Cy5-labeled end (magenta dot) with or without an EcoRI binding site (purple hexagon). **b** Reduction of Cy5 PIFE occurrence by a roadblock on L+112 but not L+112 R. **c** PIFE start timings plotted against TS-end distances. Cy5 PIFE start was timed since Cy3 vanishing from six templates, L+15 through L+512 (black squares). The timing data fit to a 1D diffusion model (blue line). **d** PIFE occurrences plotted against TS-end distances. Cy5 PIFE was counted with six templates (black squares). The occurrence data fit to the same 1D diffusion model (blue line) but not a 3D diffusion model (magenta line). All data are mean ± s.d. from three, four, or five independent experiments. bp, basepair. Source data for **b**–**d** are provided as a Source Data file.

After releasing RNA, RNAP can reach the Cy5-end of DNA through three-dimensional (3D) diffusion after dissociation from DNA or through one-dimensional (1D) diffusion (sliding or hopping) without dissociation from DNA. To examine how RNA-free RNAP reaches DNA ends, a recognition sequence of EcoRI restriction enzyme was inserted into DNA at downstream of TS (Fig. 4a). DNA-bound EcoRI blocks procession of *E. coli* RNAP,^17^ so this roadblock would obstruct 1D diffusion downward from TS, but not TS-upward 1D diffusion or 3D diffusion.

In experiments using a cleavage-lacking mutant E111Q of EcoRI,^18^ this roadblock reduces PIFE occurrence among the termination complexes when Cy5 is placed at the downstream end of L+112 (from 89 ± 2% to 25 ± 7%), but not with Cy5 placed at the upstream end of L+112 R template immobilized by the opposite, downstream end (Fig. 4a, b). As the PIFE occurrence reduction is as much as DNA cleavage inhibition by E111Q (71 ± 5%; Supplementary Fig. 5), the Cy5-ends are reached by RNAP mostly through 1D diffusion, much more often than 3D diffusion.

### Post-terminational diffusion lifetime of RNAPs

It is known that RNAP preferentially binds blunt ends of DNA, so the post-terminational retention time of RNAP or σ on short DNA molecules could be longer than the 1D diffusion lifetime, which can be measured on long DNA molecules where diffusing RNAP takes too long to reach an end. The σ-retention time (Cy5 signal duration) after termination (Cy3 signal vanishing) was measured on a long DNA of 1560 bp with 6.4% of PIFE occurrence expectation.

The post-terminational diffusion of RNAP lasts for 75 ± 5 s on this long DNA (Supplementary Fig. 6). This lifetime is clearly shorter than RNAP’s association with a short DNA of 103 bp at Cy5-end (Cy5-PIFE duration of 536 ± 82 s; Supplementary Fig. 1) or similar association of Cy5-σ holoenzyme with the short DNA (Cy5 signal duration of 588 ± 85 s; Supplementary Fig. 4). RNAP is thus apparently trapped for an extended period once it reaches the ends of DNA.

### Post-terminational diffusion coefficient of RNAPs

The 1D diffusion coefficient of post-terminational RNAPs can be estimated by the correlations that Cy5 PIFE occurs less frequently and starts later as the Cy5-end is placed further away from TS (Fig. 4c, d). Cy5 PIFE was measured with six templates, L+15, L+62, L+112, L+212, L+312, and L+512, where the distance between TS and Cy5-end of DNA (TS-downstream length) varies from 15 to 512 bp (Fig. 4a).

Termination efficiency (ranging from 31 ± 3% to 41 ± 5%) or timing (ranging from 3.8 ± 0.8 to 7.1 ± 1.3 s) is not much affected by the TS-downstream length (Supplementary Table 1; Supplementary Fig. 7). On the other hand, with increasing TS-end distance, the PIFE start delay increases from 0.6 ± 0.2 to 25 ± 9 s since RNA release (Fig. 4c), and the PIFE occurrence decreases from 91 to 47% (Fig. 4d), both supporting for 1D diffusion of RNAP on DNA after termination.

RNAP diffusion on DNA is modeled to occur with one reflecting end on the surface side and the other absorbing end on the buffer side, which is reasonable because PIFE occurrence at free ends is highly probable with short DNA molecules. This 1D diffusion model adopting the above-determined post-terminational diffusion lifetime of RNAPs fits well to the correlation of the PIFE start timing (*R*^2^ = 0.90) or the PIFE occurrence (*R*^2^ = 0.96) with the TS-end distance (Fig. 4c, d), from which the diffusion coefficient is estimated as 4.7 × 10^−4^ or 3.4 × 10^−4^ μm^2^/s, respectively (Methods section).

On the other hand, the PIFE occurrence data do not fit to a previously described 3D diffusion model^19^ (Fig. 4d). The PIFE occurrence at zero distance can be additionally estimated by the above 1D diffusion model, and 94% of complexes are estimated to retain RNAP at the time of RNA release at TS, and the remaining 6% release RNAP together with product RNA.

### Salt dependency of post-terminational diffusion

Post-terminational Cy5-PIFE occurrence with L+15 was measured with varying concentrations of NaCl from 20 to 600 mM but at fixed 2 mM MgCl_2_, which is closer to physiological conditions than 20 mM MgCl_2_ (Supplementary Fig. 8). The NaCl variation from sub-physiological 20 mM to near-physiological 150 mM does not much affect the PIFE occurrence; 78 ± 9% at 20 mM, 80 ± 4% at 50 mM, 76 ± 12% at 100 mM, and 82 ± 3% at 150 mM. However, at non-physiological 300 and 600 mM NaCl, the PIFE occurrence is reduced much to 52 ± 14% and 14 ± 12%, respectively.

When potassium glutamate replaces NaCl at 150 mM, PIFE occurrence is still 85 ± 4%, making little difference. On the other hand, MgCl_2_ reduction from 20 to 2 mM (at 20 mM NaCl) decreases PIFE occurrence to 78 ± 9%, increasing termination efficiency to 55 ± 14%. Incidentally, under a crowding condition with 15% PEG 8000, PIFE occurrence is reduced to 63% (termination efficiency of 32%), although the reduction could be larger with longer DNA.

### Transcription reinitiation by post-terminational RNAPs

We conceived that during post-terminational diffusion on DNA, RNA-free holoenzymes of RNAP could initiate transcription again when they encounter a promoter. Even core enzymes of RNAP could do so if they become adequately complemented with σ. Here ‘reinitiation’ refers to another round of promoter binding and initiation by the RNAP that has released RNA, but not fallen off DNA and diffuses on DNA rather than by 3D association of RNAP.

**Fig. 5:**
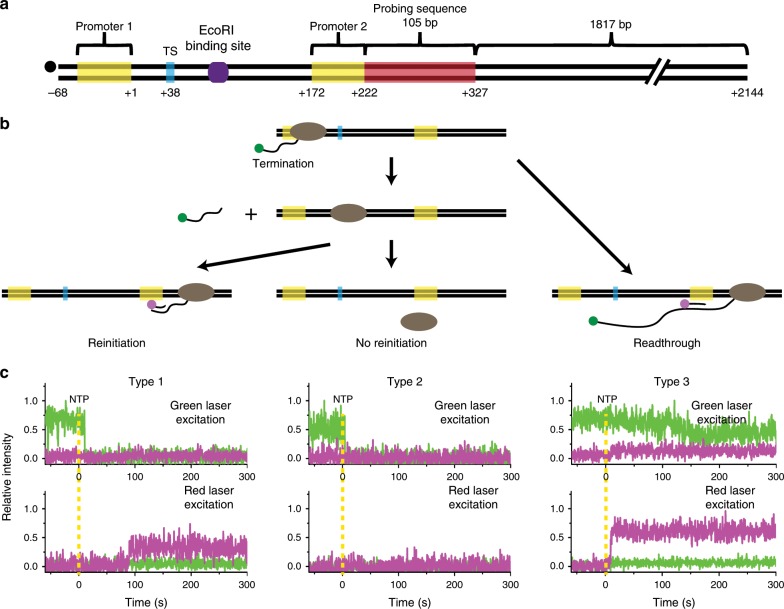
Detection of transcriptional reinitiation. **a** A DNA template contains two transcription units with a roadblock (purple hexagon) between them. Transcripts from the downstream unit have a probing target (brown box). **b** Three possible courses are reinitiation on the downstream unit after TS termination, no reinitiation after TS termination, and readthrough at TS with continued downstream transcription. **c** Three types of fluorescence time traces were observed; Cy3 vanishing with Cy5 probing (type 1), Cy3 vanishing without Cy5 probing (type 2), and Cy3 nonvanishing with Cy5 probing (type 3).

Reinitiation was examined using a template with two transcription units (Fig. 5a). L+15 containing a short transcription unit was extended downstream to contain a roadblock site and another long transcription unit, among other extensions. As explained above, short transcripts from the upstream unit can be labeled with Cy3-ApU, which reports whether termination or readthrough occurs at TS. Long transcripts from the downstream unit contain five repeating 21-bp sequences, and can be probed by a Cy5-labeled DNA oligomer complementary to the repeat sequence.

Three courses can proceed (Fig. 5b) as they were observed in different types of fluorescence time traces (Fig. 5c). First, reinitiation occurs on the downstream unit after termination from the upstream unit, as Cy5-oligomer probing signal follows Cy3-RNA signal vanishing (type 1). Second, no reinitiation occurs after TS termination due to RNAP dissociation or inactivation, as no Cy5 probing follows Cy3 vanishing (type 2). Third, readthrough occurs for continued downstream transcription, as Cy5 probing follows Cy3 nonvanishing (type 3). The relative frequencies of types 1, 2, and 3 were 0.09 ± 0.03, 0.40 ± 0.11, and 0.51 ± 0.09, respectively.

Because transcript probing was not complete, not all readthrough events were counted, and some reinitiation events were observed as type 2 instead of type 1. However, using the measured termination efficiency (33%), the probing efficiency was estimated as 52%, allowing the reinitiation (0.18) and no-reinitiation (0.31) events to be resorted (Methods section). Thus, the reinitiation portion among the termination events was 37% with a mixture of holoenzymes and core enzymes (Supplementary Table 3).

The reinitiation efficiency decreases to 19% with the above-mentioned roadblock (Supplementary Table 3), supporting that reinitiation is generated by 1D diffusion. The reinitiation efficiency increases to 55% with addition of 3 μM σ^70^ (Supplementary Table 3), which would increase holoenzyme population, suggesting that recycling core enzymes can become initiation-competent holoenzymes with σ complement while diffusing on DNA. The termination timing was little affected by the roadblock or the σ^70^ supplement. When a control experiment was performed with a template lacking the upstream transcription unit, the initial stalling complexes on the remaining downstream unit were very few in the initial stalling buffer without UTP, probably because the starting sequence is 5′-UGC.

If reinitiation occurs on the original, upstream promoter (upstream reinitiation) in addition to that on the downstream promoter (downstream reinitiation), among the RNAPs transcribing the upstream unit for the second time and subsequent times, TS readthrough yields type 1, but TS termination yields type 2. Therefore, the above-described downstream reinitiation efficiency could have been down estimated.

**Fig. 6:**
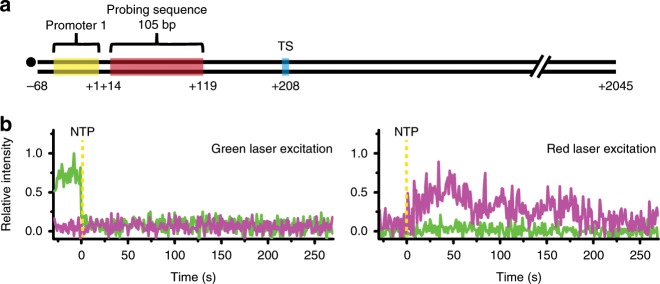
Upstream reinitiation occurring on the original promoter. **a** A DNA template has the probing target sequence (brown box) within a transcription unit located in an upstream part. **b** A representative type-1 fluorescence trace pattern, indicating reinitiation events is shown.

In order to see if upstream reinitiation occurs by backward diffusion of RNAP, another long template was constructed to have the above-described probing sequence within the upstream transcription unit but to lack any downstream unit (Fig. 6a). With supplement of 3 μM σ^70^, the relative frequencies of the three types were 0.05 ± 0.01, 0.38 ± 0.03, and 0.57 ± 0.04, respectively, as the type 1 is shown in Fig. 6b. The upstream reinitiation efficiency was estimated to be 43% (Supplementary Table 3), lower than the downstream reinitiation efficiency (55%) mentioned above.

## Discussion

This study discovered that RNA transcript is released first from transcription complex, and RNAP dissociates from DNA template much later most times in *E. coli* intrinsic termination, based on our single-molecule monitoring of fluorescent RNA transcript, DNA template, or σ factor. This sequential dissociation (94%) is much more frequent than their concurrent dissociation (6%), although it was measured with short and long linear DNA templates rather than supercoiled circular chromosomes. Considering that only 75% of elongation complexes retain σ factor, the post-terminational retention of RNAP on DNA seems to occur similarly with both core and holoenzymes. Additionally, we showed that most holoenzymes retain the σ factor after intrinsic termination.

**Fig. 7:**
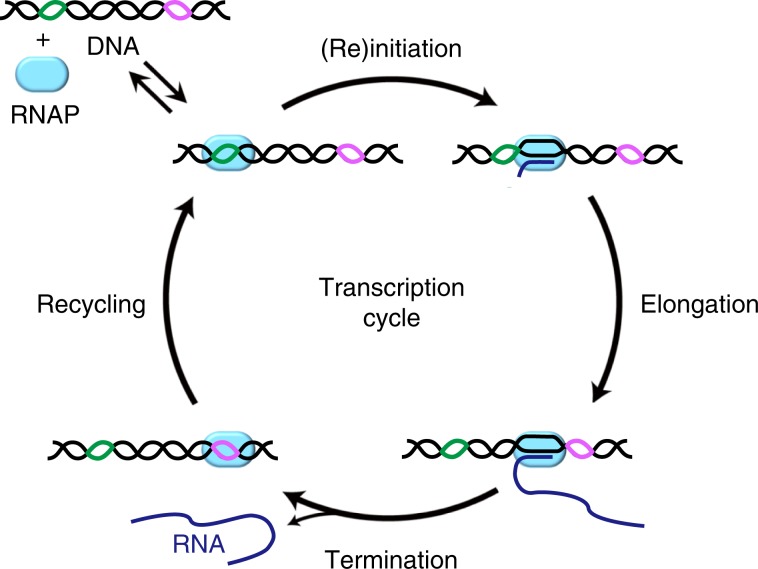
Four stages of transcription cycle. (1) Initiation or reinitiation: after binding DNA (black line) at a promoter (green line), RNAP (cyan oval) incorporates several NTPs into RNA (blue line), and advances downward clearing the promoter. (2) Elongation: RNAP further advances processively downward, resuming from occasional pause or backtrack. (3) Termination: RNAP pauses at a terminator (magenta line) to undergo conformational changes, and releases RNA product. (4) Recycling: RNAP diffuses on DNA downward and upward until it reinitiates transcription at the same or another promoter unless RNAP dissociates off DNA.

Because termination should refer to RNA release only, the subsequent retention of RNAP on DNA constitutes a previously unidentified stage. This fourth stage of transcription after the initiation, elongation, and termination stages is here termed as recycling (Fig. 7). During the recycling stage, post-terminational RNAPs diffuse on DNA one-dimensionally in downward and upward directions, until they fall off DNA.

1D diffusion of RNAP has been suggested to accelerate promoter search,^20–22^ but few experiments have directly proved it. Furthermore, RNAP diffusion on DNA was recently measured to be too short (<30 ms) to contribute to initiation kinetics under physiological conditions.^23^ This lifetime of pre-initiational RNAP on DNA is much shorter than that of post-terminational RNAP (>1 min) observed in this study, suggesting that 1D diffusion mode and conformation are distinct between the pre-initiational and post-terminational RNAPs. This study provides the first direct evidence that post-terminational RNAP’s 1D diffusion on DNA is frequent and long enough to efficiently facilitate reinitiation. This effect on reinitiation would depend on RNAP concentration and terminator–promoter distance.

RNAP’s post-terminational possession of σ and diffusion on DNA allow for transcription reinitiation, which is defined to occur by promoter binding through 1D rather than 3D diffusion of post-terminational RNAP. Reinitiation is demonstrated here to occur not only on a downstream promoter oriented in the sense direction (downstream reinitiation), but also on the original promoter located upstream of TS (upstream reinitiation) in a positive feedback fashion. Thus, post-terminational RNAP can diffuse downward and upward on DNA for reinitiation on any promoter that can be reached within the diffusion lifetime. If 1D-diffusing RNAP can flip on DNA, reinitiation at an oppositely oriented promoter (antisense reinitiation) located upstream or downstream could be possible. Furthermore, σ factor supplement is shown here to increase the reinitiation efficiency, suggesting that even core enzyme can become reinitiation-competent during the recycling stage.

Post-terminational retention and diffusion of RNAP on DNA, and transcription reinitiation competence of such RNAP during the recycling stage have several functional implications, although they have not been observed to occur in vivo yet. First, they would facilitate transcriptional burst, which has been observed for both bacterial and eukaryotic transcriptions.^24–26^ Post-terminational retention of RNAP may increase its local density and render it readily convertible into reinitiation-competent state. Forward diffusion would contribute to downstream accumulation of RNAP, although backward diffusion necessary for upstream accumulation may interfere with head-on collision with other RNAPs.

Second, simultaneous transcriptional regulation of multiple operons can be facilitated. In bacterial genomes, functionally related genes and operons are often clustered and regulated together as a regulon.^27–30^ One can hypothesize that a single RNAP molecule continually transcribes clustered operons, while diffusing on inter-operon regions of DNA chromosome.

In this work, we established a single-molecule fluorescence assay to study bacterial transcription termination dynamics. Our construction of fluorescent transcription complexes can permit single-molecule monitoring of their components in studies on transcriptional elongation, termination, recycling, and reinitiation.

## Methods

### Protein preparations

*E. coli* RNAP holoenzyme was either purchased from New England Biolabs (NEB) or a gift of Prof. Jin Young Kang at Korea Advanced Institute of Science and Technology, Korea. The Kang’s holoenzyme was reconstituted with the extensively lab-purified core RNAP and σ^70^ (RpoD) preparations. The core subunits were expressed from pVS11 encoding the α, β, β′, and ω subunits and pACYCDuet-1_Ec_rpoZ encoding the ω subunit in *E. coli* BL21(DE3). They were purified by using HiTrap IMAC (GE Healthcare) affinity, polymin P precipitation, ammonium sulfate precipitation, Bio‐Rex 70 ion exchange, and HiLoad Superdex 200 (GE Healthcare) gel filtration^31^ without a protease cleavage step. The RpoD factor was expressed with a His_6_-sumo tag from a cloned plasmid pETsumo in *E. coli* BL21(DE3) and then purified by using HiTrap IMAC HP, HiTrap Heparin HP (GE Healthcare), and HiLoad Superdex 200 columns.^32^

*E. coli* NusA was expressed with an N-terminal His_6_ tag from a cloned plasmid pNG5 in *E. coli* BL21(DE3) and purified by using HiTrap HP affinity and Superdex 200 gel filtration.^33,34^ *E. coli* NusG, another gift of Prof. Jin Young Kang, was expressed with a C-terminal His_6_ tag from a cloned plasmid pRM1160 in *E. coli* BL21(DE3) and purified by using HiTrap IMAC, HiTrap Q, and HiLoad Superdex 75 columns.^35^

EcoRI mutant E111Q was expressed with an N-terminal flag_3_ tag and a C-terminal intein tag from a cloned plasmid in *E. coli* BL21(DE3), and the mutant without the intein tag was purified by using chitin-resin column chromatography and intein cleavage.^36^ This mutant preparation has been shown to bind its cognate sequence very tightly (*K*_d_ ≈ fM) and has been used as a protein obstacle on DNA in several previous experiments.^18,37,38^

### Cy5 labeling of σ^70^ factor

*E. coli* RpoD was labeled with Cy5 at the 366th residue Cys substituting Ser, while Cys residues at the 132nd, 291st, and 295th positions had been substituted for Ser.^16^ A single-Cys derivative of RpoD was expressed with an N-terminal His_6_ tag from pRPODS366C, a gift of Prof. Robert Landick at the University of Wisconsin, USA, in *E. coli* BL21(DE3) at 25 °C and purified as described above for unmodified RpoD. The single-Cys RpoD was incubated with Cy5 maleimide mono-reactive dye (1 mM, GE Healthcare) in the storage buffer (50 mM Tris-HCl, pH 8.0, 100 mM NaCl, and 0.1 mM EDTA) overnight at room temperature. Unreacted Cy5-maleimide molecules were removed using an Amicon ultra centrifugal filter (Merck).

### Transcription templates

All transcription templates were prepared by polymerase chain reactions (PCR) using AccuPower ProFi Taq PCR premix from Bioneer, Korea with the amplification templates and primers purchased from Integrated DNA Technologies, USA. Their sequences are listed in Supplementary Tables 4 and 5. All amplification products were purified using the Cleanup kit Expin PCR SV mini from GeneAll, Korea. Details of template constructions are described in the Supplementary Methods.

### Single-molecule transcription termination experiments

DNA template (50 nM) was incubated with *E. coli* RNAP holoenzyme (20 nM, NEB), ATP, GTP, CTP (20 μM each, GE Healthcare), and Cy3-labeled ApU (250 μM, TriLink) for 30 min at 37 °C. For the experiments with Cy5-labeled σ, 20 nM core enzyme plus 1 μM Cy5-σ were used instead of holoenzyme. Quartz slides were cleaned using piranha solution to remove organic residues, incubated with (3-aminopropyl) trimethoxysilane (United Chemical Technologies), and coated with polymers by incubation in a 1:40 mixture of biotin-PEG-5000 and m-TEG-5000 (Laysan Bio). The slides were treated with 0.2 mg/ml streptavidin (Invitrogen) for 5 min, and transcription complexes were immobilized on the slides via biotin–streptavidin conjugation. They were extensively washed with an imaging buffer to remove unbound RNAPs and unimmobilized complexes.

After the readthrough molecules, the termination molecules showing Cy5 PIFE, and the termination molecules not showing PIFE were separately counted, termination efficiency was calculated as a portion of termination molecules among total molecules. PIFE occurrence was calculated as a portion of PIFE-showing termination molecules among total termination molecules. All experiments were repeated three to five times with the number of analyzed molecules (*n*) described in the Supplementary Tables 1–3, except that those with L+15 M (a negative control, *n* = 23), *his* attenuator (*n* = 92), t500 terminator (*n* = 63), or PEG8000 (*n* = 59) were performed only once.

### Single-molecule fluorescence imaging

A homemade total-internal-reflection fluorescence microscope was equipped with a 532-nm green laser (EXLSR-532-50-CDRH, Spectra-physic) for Cy3 excitation and with a 640-nm red laser (EXLSR-635C-60, Spectra-physic) for Cy5 excitation. An electron-multiplying charge-coupled camera (Ixon DV897, Andor Technology) was used as an imaging device and controlled by using a customized C# program. All experiments were performed at 37 °C with 0.05 s exposure time. The time resolution of the experiments was 0.1 s using the alternating laser excitation (ALEX) mode.

All experiments were performed with 200 μM NTP each in the oxygen scavenging buffer (10 mM Tris-HCl, pH 8.0, 20 mM NaCl, 20 mM MgCl_2_, 1 mM dithiothreitol, 5 mM 3,4-protocatechuic acid, and 100 nM protocatechuate-3,4-dioxygenase). When needed, 500 nM NusA, NusG, or both were added to the buffer. In the roadblock experiments, the immobilized samples were incubated with E111Q (1 nM) for 10 min, and DNA templates without E111Q were digested using wild-type EcoRI (10 units/μl, Beams Biotechnology). Then, the experiments were performed in the buffer containing 1 nM E111Q.

### Single-molecule transcription reinitiation experiments

The imaging buffers before and after NTP injection contained the same concentration of Cy5-labeled imager DNA (50 nM, Cy5-TGTGT GTGGT CTGTG GTGTC T-3′) to maintain the same background level. Our analysis excluded the traces showing Cy5 signal before NTP addition (nonspecific probing). Because tR2 termination occurs at 6.1 s after NTP addition on average, only Cy3 vanishing within 15 s was regarded as termination. Among these traces, we classified those with Cy5 probing within 240 s as type 1 and the others as type 2. On the other hand, those with Cy3 surviving longer than 180 s and Cy5 probing within 240 s were classified as type 3. Other traces were excluded from the data analysis. The exclusion due to Cy3 survival for 15–180 s was minor (11%). The exposure time of 0.2 s was used to reduce photobleaching.

### 1D diffusion model

Diffusion of RNAP on DNA can be modeled as 1D diffusion of a point particle with one reflecting end and one absorbing end (Supplementary Fig. 9). RNAP diffusion coefficient and RNAP retention on DNA, such as PIFE occurrence and start timing can be estimated using this model. The mean time to reach the absorbing end, *W*(*x*), is defined as a function of the distance between the reflecting end and the TS, *x*. Then,

1 $$W(x) = \tau + \frac{1}{2}\left[ {W\left( {x - \delta } \right) + W\left( {x + \delta } \right)} \right]$$

where *δ* is a step size of the random walk of the particle moving rightward or leftward every *τ* s. Because RNAP does not remain on DNA permanently, an RNAP dissociation term is added to Eq. (1).

2 $$W(x) = \tau + \frac{1}{2}\left( {1 - \frac{\tau }{{t_{\mathrm{dis}}}}} \right)\left[ {W\left( {x - \delta } \right) + W\left( {x + \delta } \right)} \right]$$

where *t*_dis_ is a dissociation time of RNAP after termination. In the limit δ→0,

3 $$W(x) - {t_{\mathrm{dis}}} = {t_{\mathrm{dis}}}D\frac{{\partial ^2}}{{\partial x^2}}W(x)$$

where *D* is a diffusion coefficient, defined as *δ*^2^/2*τ*.

When TS is positioned at the reflecting end (*x* = 0), the mean capture time does not change with *x*. When TS is positioned at the absorbing end (*x* = *L*), the particle is captured right after the particle release, generating the boundary conditions (4) and (5).

4 $$\frac{{dW}}{{dx}}_{x = 0} = 0$$

5 $$W(L) = 0$$

With these boundaries, Eq. (3) is solved as a function of *x* and the total length *L*

6 $$W(x;L) = {t_{\mathrm{dis}}}\left( {1 - \frac{{\cosh (x/l_D)}}{{\cosh (L/l_D)}}} \right)$$

where *l*_D_^2^ is equal to *t*_dis_*D*. Since *x* is fixed as 88 bp in our experiments, Eq. (6) becomes a function of the distance between TS and the absorbing end, denoted by *b*.

7 $$W\left( b \right) = {t_{\mathrm{dis}}}\left( {1 - \frac{{\cosh (88/l_D)}}{{\cosh ((88 + b)/l_D)}}} \right)$$

Diffusion coefficient of RNAP can be estimated by fitting PIFE timing data to Eq. (7).

Similarly, the probability of capture on the absorbing end, *P*(*x*), is a function of *x*. Then,

8 $$P(x) = \frac{1}{2}\left( {1 - \frac{\tau }{{t_{\mathrm{dis}}}}} \right)\left[ {P\left( {x - \delta } \right) + P\left( {x + \delta } \right)} \right]$$

9 $${\mathrm{In}}\;{\mathrm{the}}\;{\mathrm{limit}}\;{\updelta} \to 0,\;P(x) = {\mathrm{t}}_{\mathrm{dis}}D\frac{{\partial}{^2}}{{\partial}{x^2}}P(x)$$

When TS is positioned at the reflecting end, the probability of capture also does not change with *x*. Otherwise, the probability of capture is equal to the RNAP retention probability during termination at TS when TS is positioned at the absorbing end. These two conditions generate two boundary conditions.

10 $$\frac{{dP}}{{dx}}_{x = 0} = 0$$

11 $$P(L) = y_0$$

where *y*_0_ is the RNAP retention probability during termination at TS. With these boundaries,

12 $$P(x;L) = y_0\frac{{\cosh (x/l_D)}}{{\cosh (L/l_D)}}$$

Because *x* = 88 and *L* = 88 + *b*, Eq. (12) becomes a function of *b*.

13 $$P(b) = y_0\frac{{\cosh (88/l_D)}}{{\cosh ((88 + b)/l_D)}}$$

RNAP retention probability during termination at TS can be estimated by fitting PIFE occurrence to Eq. (13).

### Calculation of reinitiation efficiencies

Among the three types of fluorescence traces of Fig. 5c, type 3 uniquely represents TS-readthrough events (Fig. 5b), but does not cover all of them because transcript probing is not complete. The unprobed readthrough events are not distinguishable from nonactivated events and could not be directly counted.

On the other hand, types 1 (0.095) and 2 (0.397) together represent total TS-termination events (Fig. 5b), but their sum (0.492) is an overestimation because of incomplete probing. However, using the measured termination efficiency of 33.4%, the unprobed readthrough events (type 4) can be estimated to be 0.473, i.e., (0.492 − 0.334)/0.334. Types 3 (0.508) and 4 (0.473) together represent total readthrough events (0.981), with probing efficiency of 51.8%. Then, total readthrough and termination events become 1.473.

Due to incomplete probing, some reinitiation events must have been shown as type 2 instead of type 1. Assuming that probing efficiency was the same in readthrough and termination events, total probed and unprobed reinitiation events can be estimated to be 0.183 (=0.095/0.518), and the remaining no-reinitiation events being 0.309 = (0.492−0.183). Thus, the reinitiation portion among the total termination events is 37.2% = (0.183/0.492) with a mixture of holoenzymes and core enzymes.

### Reporting summary

Further information on research design is available in the Nature Research Reporting Summary linked to this article.

## Supplementary information

Supplementary Information

Peer Review

Reporting Summary

## Data Availability

A reporting summary for this Article is available as a Supplementary Information file. The source data underlying Figs. 4b–d are provided as a Source Data file. All other relevant data are available from the corresponding authors upon reasonable request.

## Source data

Source Data

## Code availability

The custom scripts are available from the corresponding authors upon reasonable request.
