# Supplementary material for: Transcription reinitiation by recycling RNA polymerase that diffuses on DNA after releasing terminated RNA
Source: Nat Commun. 2020 Jan 23;11:450. doi: 10.1038/s41467-019-14200-3 (PMC6978380; doi:10.1038/s41467-019-14200-3)

Supplementary Information

Transcription reinitiation by recycling RNA polymerase that diffuses on DNA after releasing terminated RNA

W. Kang et al.

Supplementary Tables

Supplementary Table 1 Termination properties of tR2 on various templates

DNA template	Termination efficiency (%)	Termination timing[a] (s)	PIFE[b] occurrence (%)	PIFE[b] start timing[c] (s)	The # of molecules analyzed (*n*) in replicated experiments
L+15	33 ± 4	6.1 ± 0.9	91 ± 5	0.6 ± 0.2	818 = 283 + 168 + 128 + 141 + 98
L+15 + lab RNAP[d]	38 ± 7	4.7 ± 0.4	95 ± 6	1.6 ± 1.1	230 = 37 + 114 + 79
L+62	31 ± 3	3.8 ± 0.5	91 ± 6	1.5 ± 0.5	217 = 86 + 78 + 53
L+112	41 ± 5	5.9 ± 0.8	89 ± 2	6.1 ± 2.4	183 = 21 + 21 + 77 + 64
L+112 + E111Q	-	4.9 ± 0.7	25 ± 7	20.7 ± 19.0	37 = 5 + 14 + 18
L+112R	-	7.1 ± 1.3	81 ± 5	9.9 ± 4.1	64 = 17 + 13 + 19 + 15
L+112R + E111Q	-	6.2 ± 1.6	84 ± 5	7.7 ± 2.3	30 = 9 + 11 + 10
L+212	34 ± 1	4.0 ± 0.9	73 ± 8	9.6 ± 3.7	134 = 43 + 35 + 23 + 19 + 14
L+312	38 ± 1	5.2 ± 1.3	71 ± 6	14.8 ± 9.5	90 = 23 + 41 + 26
L+512	36 ± 3	4.2 ± 0.9	47 ± 9	24.6 ± 8.9	404 = 100 + 58 + 75 + 101 + 70
T257/L+15	36 ± 4	25.0 ± 6.6	86 ± 2	3.6 ± 1.9	144 = 50 + 43 + 30 + 21
L+15M	none	-	none	-	23

[a] Timing of RNA release (termination) at TS measured since NTP injection

[b] Cy5 PIFE made by post-terminational RNAP

[c] Timing of Cy5 PIFE start measured since RNA release (termination)

[d] Using extensively lab-purified RNAP instead of RNAP purchased from New England Biolabs

Supplementary Table 2 Termination properties of L+15 tR2 with factors

Transcription factor	Termination efficiency (%)	Termination timing[a] (s)	PIFE[b] occurrence (%)	PIFE start timing[c] (s)	*n* in replicated experiments
NusA	58 ± 10	7.9 ± 0.2	86 ± 6	1.0 ± 0.1	236 = 86 + 78 + 72
NusG	35 ± 3	4.1 ± 0.2	90 ± 11	1.5 ± 0.5	279 = 61 + 69 + 57 + 59 + 43
NusA + NusG	58 ± 4	7.0 ± 0.8	87 ± 3	0.7 ± 1.2	223 = 93 + 69 + 61

[a] Timing of RNA release (termination) at TS measured since NTP injection

[b] Cy5 PIFE made by post-terminational RNAP

[c] Timing of Cy5 PIFE start measured since RNA release (termination)

Supplementary Table 3 Reinitiation measured with two-unit or one-unit template

DNA template	Termination timing (s)	Type 1/2/3 (%)	Probing efficiency (%)	Reinitiation efficiency (%)	*n* in replicated experiments
Two-unit	5.9 ± 1.3	9.5±2.7/39.7±10.6/50.8±9.4	51.8[a]	37.2[c]	85=19+16+19+11+20
Two-unit + σ^{70}	6.2 ± 1.3	14.1±7.3/35.0±11.8/50.9±10.1	52.0[a]	55.1[c]	86=25+25+36
Two-unit + E111Q	6.0 ± 2.3	5.6±4.9/52.5±6.5/41.9±8.8	not available	18.6[c]	59=20+19+20
One-unit + σ^{70}	7.5 ± 0.8	4.9±1.4/37.8±2.7/ 57.3±3.9	26.6[b]	42.9	122=35+32+32+23

[a] Calculated using the TS-termination efficiency of 33.4% (± 4.5%)

[b] Calculated using the TS-termination efficiency of 16.5% (± 0.9%)

[c] Reinitiation occurring on the downstream promoter

Supplementary Table 4 Template DNA sequences

Oligo name	Sequence (5' → 3')
UP8_template	TATCA AAAAG AGTAT TGACT TAAAG TCTAA CCTAT AGGAT ACTTA CAGCC ATCGA ACAGG CCTGC TGGTA ATCGC AGGCC TTTTT ATTTG GGGGA GAGGG AAGTC ATGAA AAAAC TAACC TTTGA AATTC GATCT CCAGG ATCCA CCACC
UP8M_template	TATCA AAAAG AGTAT TGACT TAAAG TCTAA CCTAT AGGAT ACTTA CAGCC ATGGC CTGCT GGTGA CTGAC TGACT GACTG AC
t500_template	TATCA AAAAG AGTAT TGACT TAAAG TCTAA CCTAT AGGAT ACTTA CAGCC ATCCC AAAGC CCGCC GAAAG GCGGG CTTTT CTGTT TCTGG GCGGT GAAGT CATGA AAAAA CTAAC CTTTG AAATT CGATC TCCAG GATCC ACCAC C
his_template	TATCA AAAAG AGTAT TGACT TAAAG TCTAA CCTAT AGGAT ACTTA CAGCC ATCCG AAAGC CCCCG GAAGA UGCAU CUUCC GGGGG CUUUU UUUUU TGGGC GGTGA AGTCA TGAAA AAACT AACCT TTGAA ATTCG ATCTC CAGGA TCCAC CACC
UP8_template_2	GCGAG ATTAC CATTA AGTGA ATTCG AAAAA AGCAC GCTAC CGCCC CAGGC GGTGG TGGAT CCTGG AGATC GAATT TCAAA GGTTA GTTTT TTCAT GACTT CCCTC TCCCC CAAAT AAAAA GGCCT GCGAT TACCA GCAGG CCTGT TCGAT GGCTG TAAGT ATCCT ATAGG TTAGA CTTTA AGTCA ATACT CTTTT TGATA
additional_part	pAATTC TTACA ATTTA GACCC TAATA TCACA TCAGA CACTA ATTGC CTCTG CCAAA ATTCT GTCCA CAAGC GTTTT AGTTC GCCCC AGTAA AGTTG TCAAT AACGA CCACC AAATC CGCAT GTTAC GGGAC TTCTT ATTAA TTCTT TTTTC GTGGG GAGCA GCGGA TCTTA ATGGA TGGCG CCAGG TGGTA TGGAA GC
additional_part_2	pGGGCT GAAAG TAGCG CCGGG TAAGG TACGC GCCTG GTATG GCAGG ACTAT GAAGC CAATA CAAAG GCTAC ATCCT CACTC GGGTG GACGG AAACG CAGAA TTATG GTTAC TTTTT GGATA CGTGA AACAT GTCCC ATGGT AGCCC AAAGA CTTGG GAGTC TATCA CCCCT AGGAC ACACA AGACA CCACA AGCTT AGACC
DNA_splint	TGTGA TATTA GGGTC TAAAT TGTAA GAATT GCGAG ATTAC CATTA AGTGA ATTCG AAAAA
DNA_splint_2	GCGTA CCTTA CCCGG CGCTA CTTTC AGCCC GCTTC CATAC CACCT GGCGC CATCC ATTAA
reinitiation_part_1B	pTAATA TCACA TCATT AGACA CTTAT CAAAA AGAGT ATTGA CTTAA AGTCT AACCT ATAGG ATACT TACAG CCTGC AGACA CCACA GACCA CACAC AAGAC ACCAC AGACC ACACA CAAGA CACCA CAGAC CACAC ACAAG ACACC ACAGA CCACA CACAA GACAC CACAG ACCAC ACACA AGACA CCACA AGCTT AGACC
DNA_splint_reinitiation	AGTGT CTAAT GATGT GATAT TAGCG AGATT ACCAT TAAGT GAATT CGAAA AA
T257/L+15_template_1	TATCA AAAAG AGTAT TGACT TAAAG TCTAA CCTAT AGGAT ACTTA CAGCC ATCGA ACAGG CCTCA AACAA AAGAA TGGAA TCAAA GTTAA CTTCA AAATT AGACA CAACA TTGAA GATGG AAGCG TTCAA CTAGC AGACC ATTAT CAACA AAATA CTCCA ATTGG CGATG GCCCT GTCCT TTTAC CAGAC AACCA TTACC
T257/L+15_template_2	TTTAC CAGAC AACCA TTACC TGTCC ACACA ATCTG CCCTT TCGAA AGATC CCAAC GAAAA GAGAG ACCAC ATGGT CCTTC TTGAG TTTGT AACAA CAGGC CTGCT GGTAA TCGCA GGCCT TTTTA TTTGG GGGAG AGGGA AG
Original_reinitiation_1	TATCA AAAAG AGTAT TGACT TAAAG TCTAA CCTAT AGGAT ACTTA CAGCC ATCGA ACAGG CCTAG ACACC ACAGA CCACA CACAA GACAC CACAG ACCAC ACACA AGACA CCACA GACCA CACAC AAGAC ACCAC AGACC ACACA CAAGA CACCA CAGAC CACAC ACAGC AGGAT TAAGA AGCCA ATACA AAGGC TAC
Original_reinitiation_2	pATCCT CACTC GGCAG AUAUG ACAAU ACAGA GGCCT GCTGG TAATC GCAGG CCTTT TTATT ACACA CAAGA CACCA CAAGC TTAGA CC
Original_reinitiation_splint	TCTGT ATTGT CATAT CTGCC GAGTG AGGAT GTAGC CTTTG TATTG GCTTC TTAAT CCTGC

Supplementary Table 5 Primer sequences

Oligo name	Sequence (5' → 3')
forward_primer_biotin	Biotin-TATCA AAAAG AGTAT TGACT TAAAG TC
reverse_primer_biotin	Biotin-GCGAG ATTAC CATTA AGTGA A
forward_primer_Cy5	Cy5-TATCA AAAAG AGTAT TGACT TAAAG TC
reverse_primer_L+15	Cy5-CTTCC CTCTC CCCCA AATAA AAAG
reverse_primer_L+15M	Cy5-GTCAG TCAGT CAGTC AGTCA CCAGC AG
reverse_primer_L+62	Cy5-GGTGG TGGAT CCTGG AGATC G
reverse_primer_L+112	Cy5-GCGAG ATTAC CATTA AGTGA A
reverse_primer_L+212	Cy5-GACAA CTTTA CTGGG GCGAA CTAAA AC
reverse_primer_L+312	Cy5-GCTTC CATAC CACCT GGCGC CATCC AT
reverse_primer_L+512	Cy5-GGTCT AAGCT TGTGG TGTCT TGTGT GT
lambda_forward_primer	GTTTT CTGGG TTGGT
lambda_reverse_primer	GGCGG GTTTT GTTTT
forward_primer_extension	ACTAT CTATT CTCCC ATCTA TCAAA AAGAG TATTG ACTTA AAGTC
forward_primer_biotin_α	Biotin-ACTAT CTATT CTCCC ATC

Supplementary Methods

DNA template L+15 was prepared using UP8_template, forward_primer_ biotin, and reverse_primer_L+15; L+15M prepared using UP8M_template, forward_primer_biotin, and reverse_primer_L+15M; L+62 prepared using UP8_template, forward_primer_biotin, and reverse_primer_L+62; L+112 prepared using UP8_template_2, forward_primer_biotin, and reverse_primer_ L+112; and L+112R prepared using UP8_template_2, forward_primer_Cy5' and reverse_primer_biotin.

Template for T257/L+15 was prepared using T257/L+15_template_1, T257/L+15_template_2, forward_ primer_biotin, and reverse_primer_L+512. Template for phage ϕ82 t500 terminator was prepared using t500_template, forward_ primer_biotin, and reverse_primer_L+62; and template for *E. coli his* operon attenuator using his_template, forward_primer_biotin, and reverse_primer_L+62.

For L+212 and L+312 constructions, L+112 and additional_part_1 were annealed with DNA_splint_1 by cooling from 90°C to 30°C for 120 min in the annealing buffer (10 mM Tris-HCl, pH 8.0, with 50 mM NaCl), ligated using T4 DNA ligase 2 purchased from NEB, and used for amplification reactions. L+212 was prepared using forward_primer_ biotin and reverse_ primer_L+212; L+312 using forward_primer_biotin and reverse_primer_ L+312. For L+512 construction, L+312 and additional_ part_2 were annealed with DNA_splint_2 and the same ligation reaction was repeated. L+512 was prepared using forward_primer_biotin and reverse_primer_L+512.

For reinitiation detection and σ retention time estimation, long_tail template with a HindIII recognition sequence was prepared using lambda DNA (NEB), lambda_forward_primer, and lambda_reverse_primer. To construct the upstream part of reinitiation template, L+112_α was prepared with UP8 template_2, forward_primer_extension, and reverse_primer_L+112. L+112_α and reinitiation_part_1B were annealed with DNA_splint_reinitiation by cooling from 90 to 30 °C for 120 min in the annealing buffer and the same ligation. To construct the upstream part of original reinitiation template, Original_reinitiation_1 and Original_reinitiation_2 were annealed with Original_reinitiation_splint by cooling from 90°C to 30°C for 120 min in the annealing buffer and the same ligation. Long_tail DNA, the upstream part of reinitiation template, and L+512 were each digested with HindIII (NEB) for one h at 37 °C in the CutSmart™ buffer (NEB). After that, HindIII was deactivated for 20 min at 80 °C.

Long_tail DNA were annealed with cleaved L+512 or the upstream part of reinitiation template by the same protocol as above and used for amplification reaction. L+lambda was prepared using forward_primer_biotin and lambda_forward_primer. DNA template for reinitiation detection was prepared using forward_primer_biotin_α and lambda_reverse_ primer. The DNA template for probing the reinitiation at the original promoter was prepared using forward_primer_biotin_α and lambda_reverse_ primer.

Supplementary Figures

Supplementary Fig. 1 RNAP·DNA complex duration at the DNA end after RNA release. We measured Cy5 PIFE survival time after the RNA release (*n* = 120), and the distribution was fitted to a single exponential function to obtain the RNAP's retention time of 536 ± 83 s.

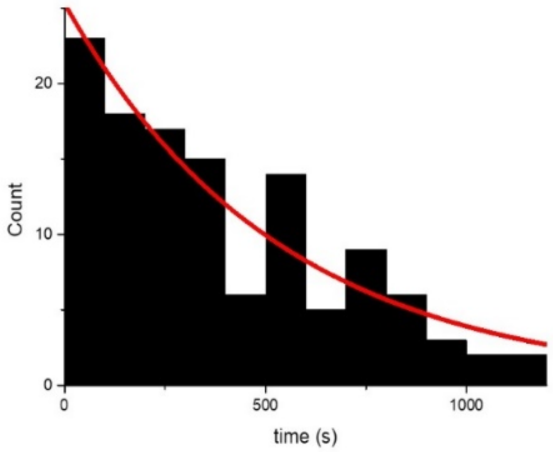

Supplementary Fig. 2 Cy5 photobleaching time. To measure Cy5 survival time, we performed single-molecule imaging without NTP injection (*n* = 122). About 60% of the molecules survived for longer than 1200 s after single-molecule imaging started, from which the photobleaching time of Cy5 was estimated as 2350 s.

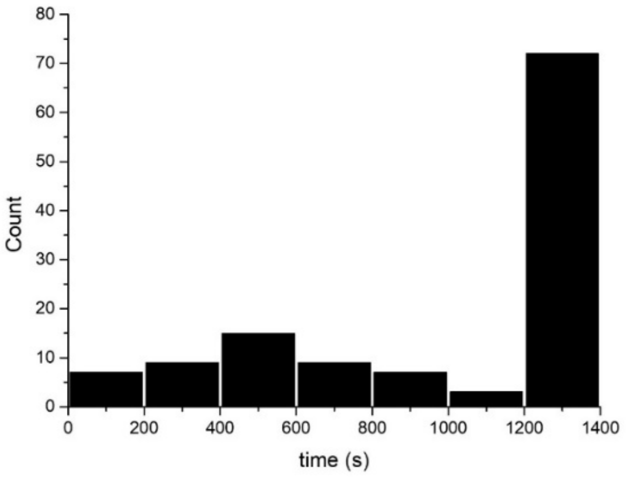

Supplementary Fig. 3 Sigma retention. We estimated the percentage of transcription complex containing σ^{70} by comparing the number of Cy3-Cy5 colocalized spots between the cases of Cy5-end of DNA and those of Cy5-σ. The Cy5 labeling efficiencies of DNA (99%) and σ^{70} (105%) were similar. When Cy5 was labeled at the DNA end, 46 ± 10% of Cy3 signal was colocalized with Cy5. When Cy5 was labeled on σ^{70}, 35 ± 6% of Cy3 signal was colocalized with Cy5. In the figures, the green circles, red dots, and black dots indicate all Cy3 spots identified, Cy3 spots colocalized with Cy5 signal, and the Cy3 spots non-colocalized with Cy5 signal, respectively. From these results, we estimated that 75% (34.5 / 45.8) of elongation complex has σ^{70}.

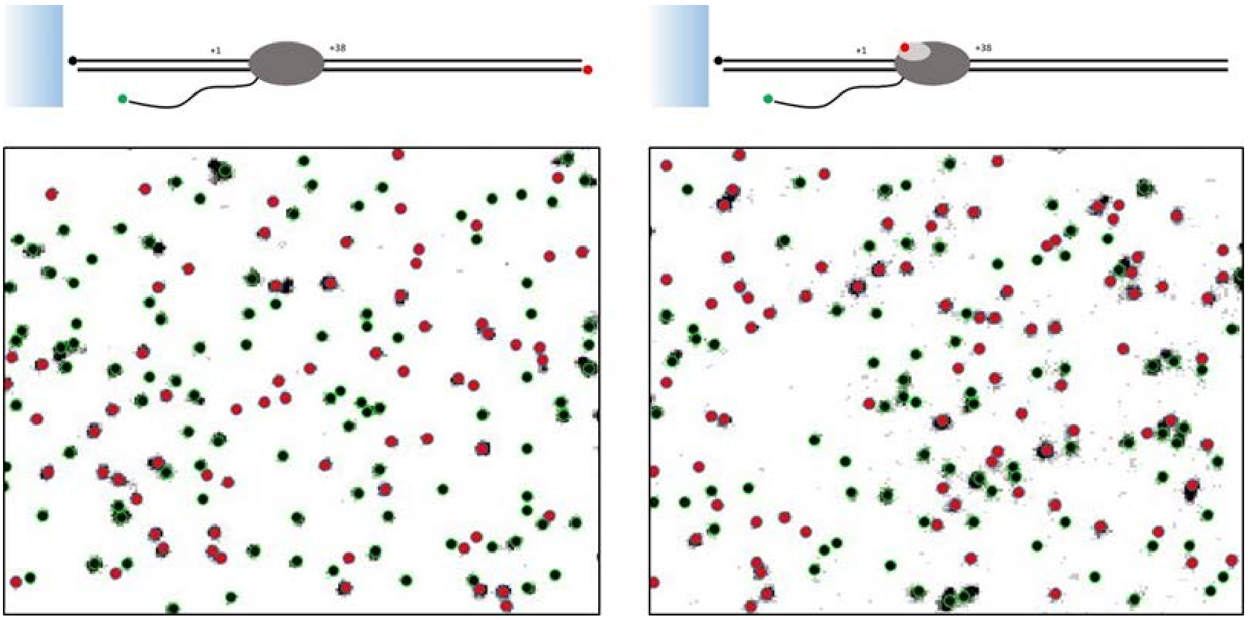

Supplementary Fig. 4 Sigma factor lasting time at the DNA end after RNA release. We measured Cy5 signal vanishing time after NTP addition in termination complexes of Fig. 1F template (n = 26).

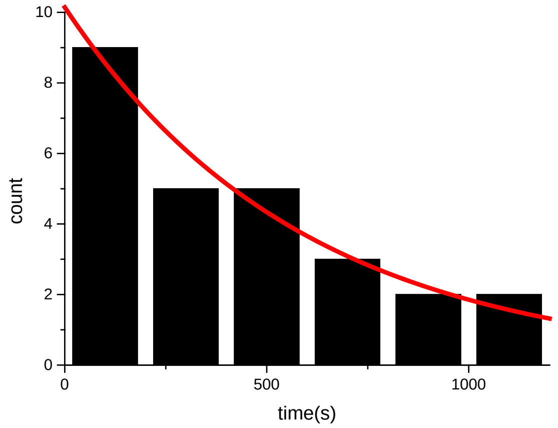

Supplementary Fig. 5 EcoRI E111Q inhibits the cleavage of DNA by EcoRI. Using the L+112 DNA template labeled with Cy5, we measured the cleavage inhibition efficiency of 1 nM E111Q. We counted the number of Cy5 spots before (left) and at 10 min after wild-type EcoRI injection (right) without (top) and with E111Q incubation for 5 min (bottom) before starting the imaging. The number of spots was decreased by 86 ± 2% without E111Q incubation. On the other hand, the number was decreased by 26 ± 4% with E111Q incubation. From these data, we estimated the cleavage inhibition efficiency of E111Q as 71 ± 5%.

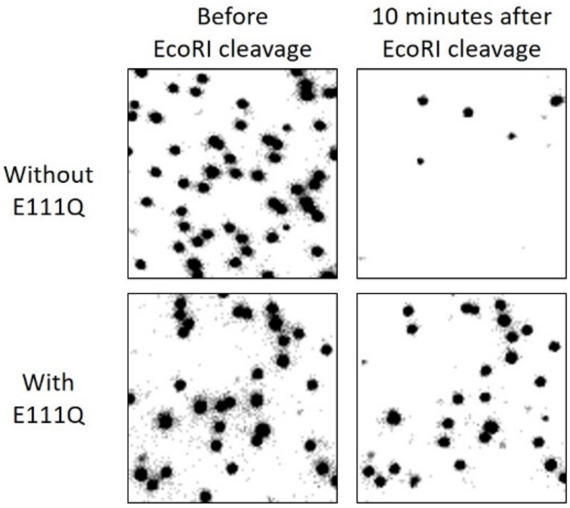

Supplementary Fig. 6 Diffusion time of RNAP on a long DNA template. We prepared a transcription complex with Cy5-labled σ^{70} on a 1560-bp DNA template (L+lambda), and measured the Cy5 signal vanishing time after termination (*n* = 30).

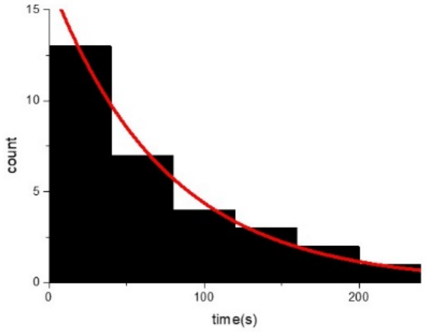

Supplementary Fig. 7 Termination time of various templates. We measured the Cy3 signal vanishing time after NTP addition for L+15 (*n* = 270, **a**), L+62 (*n* = 52, **b**), L+112 (*n* = 50, **c**), L+112R (*n* = 51, **d**), L+212 (*n* = 21, **e**), L+312 (*n* = 24, **f**), L+512 (*n* = 133, **g**), L+112 with E111Q (*n* = 46, **h**), and L+112R with E111Q (*n* = 30, **i**). The distributions were fitted to a single-exponential decay function, and the fitted decay times are summarized in Supplementary Table S1.

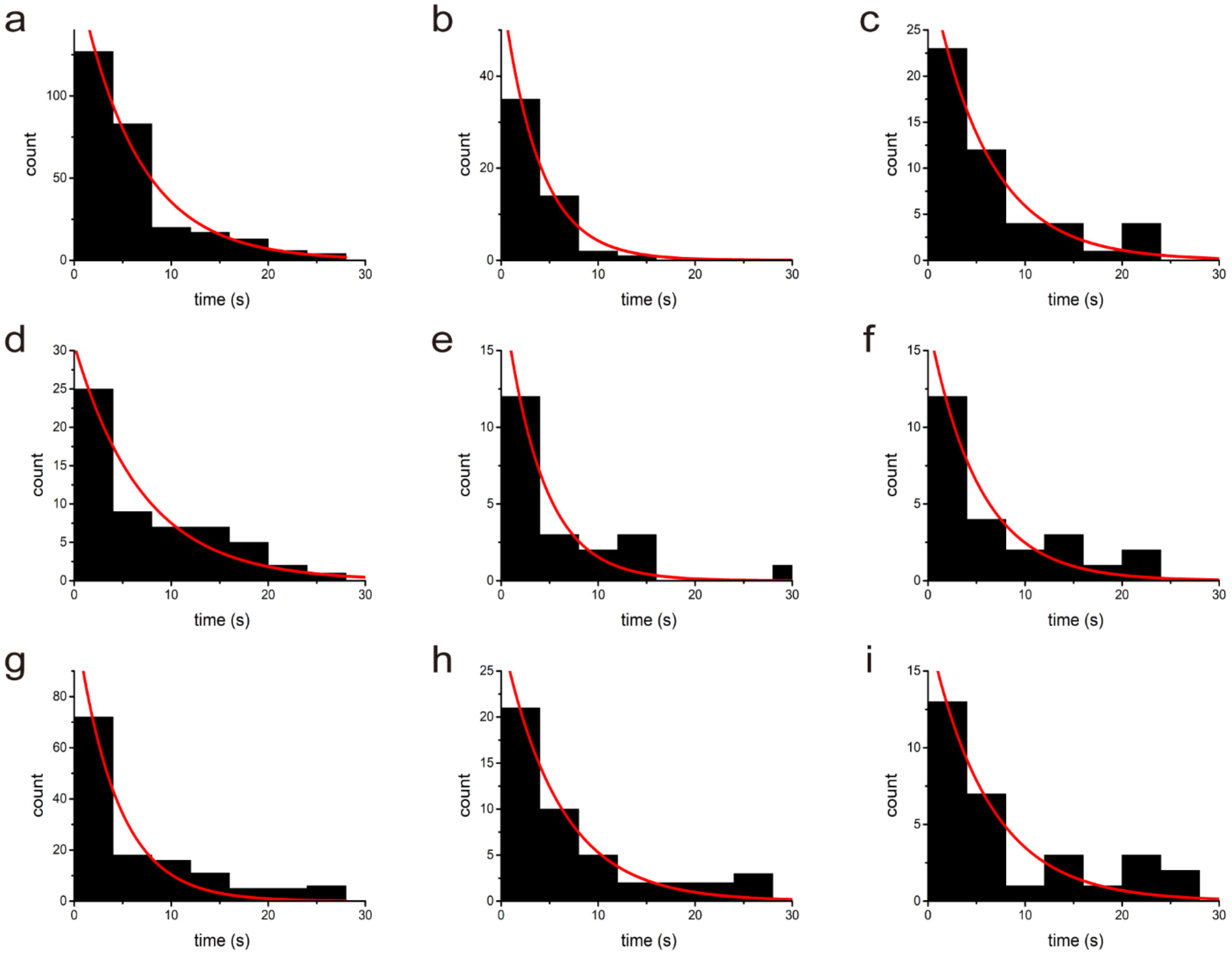

Supplementary Fig. 8 Salt dependency of post-terminational RNAP retention. The Cy5-PIFE occurrence with L+15 template was measured at varying concentrations of NaCl (from 20 to 600 mM) but at fixed 2 mM $MgCl_2$ (red circulars). It was also measured at 20 mM NaCl and 20 mM $MgCl_2$ (black square).

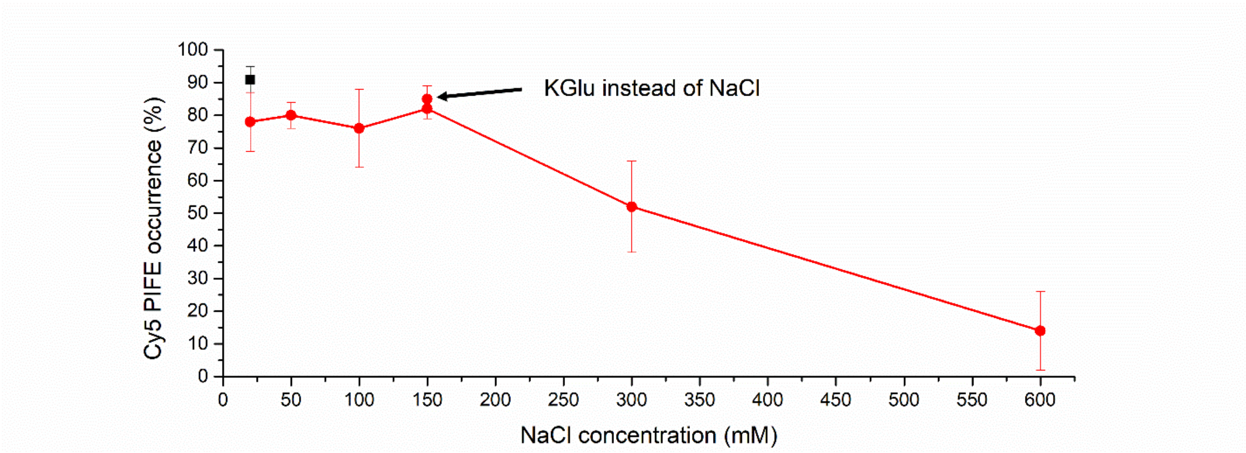

Salt (plus 2 mM $MaCl_2$)	Termination efficiency (%)	PIFE occurrence (%)	The # of molecules analyzed (*n*) in replicated experiments
NaCl 20 mM	55 ± 14	78 ± 9	117 = 13 + 31 + 73
NaCl 50 mM	47 ± 4	81 ± 5	122 = 38 + 23 + 14 + 47
NaCl 100 mM	44 ± 10	76 ± 12	122 = 37 + 37 + 48
NaCl 150 mM	44 ± 7	82 ± 3	286 = 30 + 70 + 61 + 67 + 58
NaCl 300 mM	43 ± 8	52 ± 14	98 = 14 + 25 + 19 + 13 + 27
NaCl 600 mM	43 ± 6	14 ± 12	48 = 10 + 16 + 22
KGlu 150 mM	51 ± 4	86 ± 4	54 = 15 + 19 + 20

Supplementary Fig. 9 RNAP one-dimensional diffusion model. RNAP diffusion is simplified by one-dimensional diffusion with one reflecting end and another absorbing end. TS, termination site.

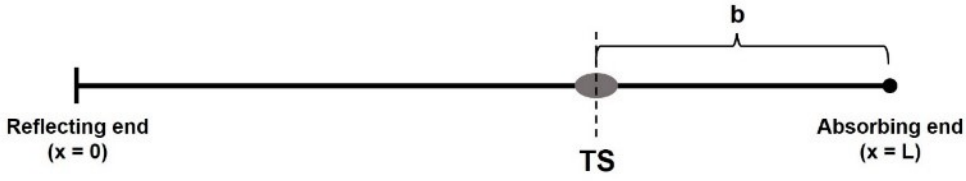

Supplement: Supplementary file 1 — Supplementary Information [file 41467_2019_14200_MOESM1_ESM.pdf]
