# Supplementary material for: Transcription reinitiation by recycling RNA polymerase that diffuses on DNA after releasing terminated RNA
Source: Nat Commun. 2020 Jan 23;11:450. doi: 10.1038/s41467-019-14200-3 (PMC6978380; doi:10.1038/s41467-019-14200-3)
Supplement: Supplementary file 2 — Peer Review [file 41467_2019_14200_MOESM2_ESM.pdf]

Editorial Note: Parts of this peer review file have been redacted as indicated to remove third-party material where no permission to publish could be obtained.

Reviewers' comments:

Reviewer #1 (Remarks to the Author):

Manuscript by Kang et al aims to have "examined the temporal order of <transcription termination complexes> dissociation and their post-terminational fates in E. coli intrinsic termination" by means of "single-molecule fluorescence measurements". While I find the findings potentially interesting, the design of the experiments neglected to replicate some of the essential elements of intrinsic termination in E. coli. As specified below, some major improvements are needed to actually achieve the stated goals.

General issues:

1. The main template is extremely short and contains just enough transcribed sequence to form a short RNA hairpin, acting as an intrinsic terminator. Not only this design fails to recapitulate the spacial parameters of the vast majority of E. coli transcription units, it limits transcriptional events to the promoter-proximal area prone to in vitro artifacts. Moreover, all the DNA templates used do not recapitulate the large, circular, and torsionally stressed genomic DNA of E. coli, but instead represented by short, linear, and relaxed DNA fragments. Such templates are a well-known source of artifacts due to RNA polymerase molecules binding to, and initiating and terminating transcription on template ends, as well as template switching, which the authors failed even to mention, let alone consider in the report.

2. It is true that the holoenzyme is necessary and sufficient to carry out the act of promoter-specific initiation of transcription, and that intrinsic termination in vitro can be carried out in factor-independent fashion. However, since the stated goal of the report is to elucidate the details of transcription in E. coli, omission of the general elongation factors NusA, NusG, and DksA, as well as cleavage factors GreA and GreB renders this experimental design irrelevant. These factors affect the rate of transcription elongation and response to pause and termination signals; moreover, some of them greatly influence transcription initiation efficiency and promoter clearance (GreA/B, DksA), while others directly compete with initiation factor RpoD for binding to early elongation complex (e.g. NusG). There are other factors which aid elongation complex in clearing roadblocks, dissociation of RNA polymerase and RNA from the template (Mfd, Rho, UvrD, etc), but unlike the first subset, these factors are present in the cell in sub-stoichiometric amounts (relative to RNA polymerase) and their omission may be overlooked in a generalized study. Factors like NusG and NusA are present essentially in every elongation complex throughout the cycle, and for all practical experimental purposes can be considered subunits of the elongation complex.

3. The choice of the experimental design which is rather speculatively based on "appearance" and "disappearance" of variously labeled RNA signals seems particularly ill-suited for the stated goal of elucidating the temporal order of elongation complex dissociation at intrinsic terminators. 6 years had passed since publication of the report by Green and colleagues (Ref 21), describing direct tracking of initiation and elongation RNA polymerases complexes along template by means of single-molecule fluorescence microscopy. Given the fact that, if needed, every component of the complex can be fluorescently labeled (core, sigma factor, and lacking from this work NusA, NusG, GreA, etc), the fate of transcription complexes before, during, and after termination can be evaluated directly and transparently. Instead the authors chose an indirect method, which appears to be a significant step backwards compared to the single-molecule tracking of elongation complexes.

4. The effects of macromolecular crowding on cellular processes have been assumed to be significant for quite some time now, but recently a rather detailed and convincing experimental investigation into these effects (as they pertain to E. coli transcription) have been published (Chung et al. The effect of macromolecular crowding on single-round transcription by Escherichia coli RNA polymerase. Nucleic Acids Res. 2019 Feb 20;47(3):1440-1450. doi: 10.1093/nar/gky1277.). In addition to the quite

dramatic effects on transcription efficiency, macromolecular crowding mimicking the conditions within the cell would have significantly affected diffusion-limited processes.

Technical issues:

1. The report is based on the commercially sourced E. coli RNA polymerase core and holoenzymes. The manufacturer (NEB) makes no claims regarding the exact composition or specific activities of these preparations, other than the ability to initiate transcription with or without additional supplementation with sigma factor, and the lack of nuclease activities. Common contaminants of RNA polymerase preparations (transcription factors, RNA and DNA helicases, etc) will drastically affect the properties of the transcription complex, and, being usually present in substoichiometric amounts, will introduce unknowable error/bias into the data. These considerations are the main reason for the extensive purification of the enzymes for in vitro experiments in general, and for the single-molecule ones in particular. Furthermore, if core and holoenzyme are to be compared in the same set of experiments, holoenzyme should be assembled using the same core and individually purified sigma subunit, as opposed to being procured independently and thus subject to additional variation between the preparations.

2. The provenance of the EcoRIQ111 is not described at all, which makes impossible to interpret the data concerning its “roadblocking” effect (depending on the expression and purification protocol, this protein may contain variable amounts of activity, DNA and/or associated proteins).

3. Purification of RpoD (sigma70) for fluorescent labeling is described in enough detail to conclude that a simple combination of Ni-affinity and heparin-affinity steps is not sufficient for the purposes of this work. Thus purified, RpoD preparations are contaminated with holoenzyme, and various sigma-binding proteins. Furthermore, this inadequately purified sigma was simply mixed with core to obtain holoenzyme in 50-fold excess. No attempt was made to purify the assembled holoenzyme (by combination of size-exclusion and ion-exchange chromatography); this in turn made unknowable the fraction of assembled holoenzyme, and created a great disparity between the commercial holoenzyme preparation (containing approximately 1 molecule of RpoD per complex) and in house assembled holoenzyme, which contained 50 times more. 50-fold excess of free sigma subunit present in sigma-dissociation experiments would drastically affect the sigma retention compared to the cellular conditions, where sigma factors were found to exist in sub-stoichiometric (relative total RNA polymerase).

4. When the authors examined the directionality of post-termination RNA polymerase 1-D diffusion, they employed DNA template L +112R attached to the cover slip via its downstream part as opposed to the upstream (non-transcribed) part in all other cases they studied. How such would this affect Cy5 PIFE fluorescence if RNA polymerase not only reached the end of the template but also the cover slip?

5. In the case of the double promoter setup for re-initiation probing, the authors did not explain how they ruled out a possibility that the second RNA polymerase occupied the downstream promoter and that they observed initiation from the already occupied promoter when all nucleotides were present.

Minor:
The treatment of transcription termination in the introduction makes a significant omission of the key mechanistical paper (PMID: 18158897). This paper explains the universal molecular pathway leading to intrinsic termination, which occurs via conformation changes of RNAP and does not involve any forward translocation or RNA shearing proposed by Larson et al (ref.9). Another obvious omission is the paper describing the original observation of persistent sigma70 retention during elongation (PMID: 11525730). That paper also demonstrated facilitated recycling by such a subpopulation of sigma70-retaining elongation complexes. Both papers must be properly cited and discussed in the revised manuscript.

Reviewer #2 (Remarks to the Author):

The authors present experimental evidence for the maintenance of an RNA polymerase/DNA complex subsequent to RNA transcript release during termination. Using a variety of template lengths and the occurrence of fluorescence enhancements, they show that this complex likely undergoes 1D diffusion on the DNA in the absence of any RNA. Finally, they show that this diffusing complex is capable or re-initiation on a downstream promoter. These observations run counter to transcriptional dogma for bacteria, and as such, will be of interest to a wide range of researchers. While potentially significant, there are significant concerns that should be addressed prior to recommending publication.

(1) I wonder if 38nt is a well defined EC. It would be reassuring to see the same behavior with a longer transcriptional unit.

(2) Salt! The experiments are being performed at 20 mM NaCl which is quite low compared to typical in vitro transcription experiments (~150 mM) and to estimates of in vivo conditions. This is of critical importance as the maintenance of the polymerase/DNA interaction should be quite salt dependent. Here I am specifically referencing both the concentration of salt, but also the identity (i.e. NaCl vs. KGlu). Far beyond reporting the conditions, an actual titration of salt concentration should most certianly be performed. At what concentration does retention of RNAP after termination become disfavored and how does this concentration compare to estimates of in vivo salt concentrations?

(3) Fig1E, what is the jump from 0.5 to 1 in the red laser excitation?
This figure would benefit greatly from a listing of what each (D,E, and G) are referring to. Some kind of subtitles for example (D: readthrough, E: termination)
Also some quantification of how often each behavior is observed would also be helpful in the figure itself (as described in the text).

(4) Line 122: Under no cases did sigma dissociate before the RNA? Do the authors meant that IF sigma was observed in the stalled elongation complex, it remained until after RNA dissociation? Or do they mean that they ALWAYS observed sigma in complexes where they observed RNA? This needs be specified. (I imagine it is the former as even the studies showing retention of sigma show ~30% dissociation upon entering the elongation complex I believe).

(5) Type 2 under reinitiating section. No (re)-initiation, but what is the evidence for dissociation of RNAP? It seems rather than although no secondary RNA is produced, the labeling strategy makes it ambiguous as to whether RNAP is still present.

(6) Also, in the case of 1D diffusion, doesn’t one also expect to see cases of re-initiation on Promoter 1? This could be seen by the re-occurrence of a green signal. If this is not observed normally, certainly in the presence of the EcoR1 barrier, the model predicts it?

(7) line 226: what is the evidence that this mechanism is “dramatically” faster compared to de novo transcription initiation? Won't this depend on the free concentration of RNAP and the distance between terminators and promoters?

(8) I don’t understand how (re)-initiation would contribute to transcriptional bursts as it would have to diffuse backwards against the stream of other RNAPs. Unless the authors mean that the polymerase would diffuse within a loop to re-encounter the first promoter, but I don’t understand how a loop would interact with 1D diffusing RNAPs.

Response to the Comments of Reviewer #1:

Manuscript by Kang et al aims to have "examined the temporal order of <transcription termination complexes> dissociation and their post-terminational fates in E. coli intrinsic termination" by means of "single-molecule fluorescence measurements". While I find the findings potentially interesting, the design of the experiments neglected to replicate some of the essential elements of intrinsic termination in E. coli. As specified below, some major improvements are needed to actually achieve the stated goals.

1. The main template is extremely short and contains just enough transcribed sequence to form a short RNA hairpin, acting as an intrinsic terminator. Not only this design fails to recapitulate the spatial parameters of the vast majority of E. coli transcription units, it limits transcriptional events to the promoter-proximal area prone to in vitro artifacts. Moreover, all the DNA templates used do not recapitulate the large, circular, and torsionally stressed genomic DNA of E. coli, but instead represented by short, linear, and relaxed DNA fragments. Such templates are a well-known source of artifacts due to RNA polymerase molecules binding to, and initiating and terminating transcription on template ends, as well as template switching, which the authors failed even to mention, let alone consider in the report.

[Our response] To be included in the revision, additional PIFE (protein-induced fluorescence enhancement) experiments were performed with a newly constructed long DNA template, denoted by T257/L+15, that contains a 257-bp transcription unit in place of 38-bp unit of the main template L+15. Cy5 PIFE occurrence on the longer T257/L+15 is 86 ± 2%, similar to 91 ± 5% on the shorter L+15. Accordingly, the post-terminational PIFE occurrence is not limited to the promoter-proximal termination. These new results are added to the Results section (page 5, lines 124-127).

We understand that linear DNA templates could sometimes permit either transcription initiation at 3'-overhang ends or elongation after template switching at blunt ends, as especially observed with phage T7, T3 or SP6 RNA polymerase. In our experiments with *E. coli* RNA polymerase, all templates have blunt ends, which would suppress the aberrant end-initiation. Furthermore, their two 5' ends are each labeled with biotin or Cy5 dye, which would suppress the aberrant end-elongation.

DNA template immobilization is required in our experimental scheme, but immobilization of supercoiled circular DNA is technically impractical, so we would like to discuss this limitation in the Discussion section (page 9, lines 259-260).

2. It is true that the holoenzyme is necessary and sufficient to carry out the act of promoter-specific initiation of transcription, and that intrinsic termination in vitro can be carried out in factor-independent fashion. However, since the stated goal of the report is to elucidate the details of transcription in E. coli, omission of the general elongation factors NusA, NusG, and DksA, as well as cleavage factors GreA and GreB renders this experimental design irrelevant. These factors affect the rate of transcription elongation and response to pause and termination signals; moreover, some of them greatly influence transcription initiation efficiency and promoter clearance (GreA/B, DksA), while others directly compete with initiation factor RpoD for binding to early elongation complex (e.g. NusG). There are other factors which aid elongation complex in clearing roadblocks, dissociation of RNA polymerase and RNA from the template (Mfd, Rho, UvrD, etc.), but unlike the first subset, these factors are present in the cell in sub-stoichiometric amounts (relative to RNA polymerase) and their omission may be overlooked in a generalized study. Factors like NusG and NusA are present essentially in every elongation complex throughout the cycle, and for all practical experimental purposes can be considered subunits of the elongation complex.

[Our response] The stated goal of this study was to determine the temporal order of DNA and RNA dissociations from RNA polymerase (holoenzyme or core enzyme) during intrinsic termination, while we did not yet aim to monitor all the other transcription factors or to examine their effects on

the dissociation order towards elucidating the details of transcription.

For this revision, however, we examined the effects of NusA and NusG. The post-terminational Cy5 PIFE occurrence is 86 ± 6% with NusA, 90 ± 11% with NusG, and 87 ± 3% with both NusA and NusG, all similar to the previous 91 ± 5% without NusA or NusG. Accordingly, the presence of NusA or NusG little affects the RNAP retention. These new results are shown in the Supplementary Table 2, and described in the Results section (page 4, lines 115-120). The NusA and NusG preparations are newly described in the Methods section (page 11, lines 325-330).

NusA was expressed with an N-terminal His_6 tag from a cloned plasmid pNG5 in *E. coli* BL21(DE3) and purified by using HiTrap HP affinity chromatography followed by gel filtration on Superdex 200, as previously described in *J. Biol. Chem*. (2007) 282, 19020–19028 and *Cell* (1995) 81, 341-350. Gel separations of the column fractions are shown below.

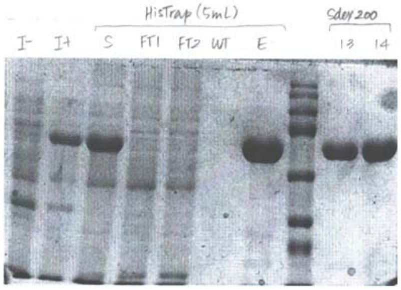

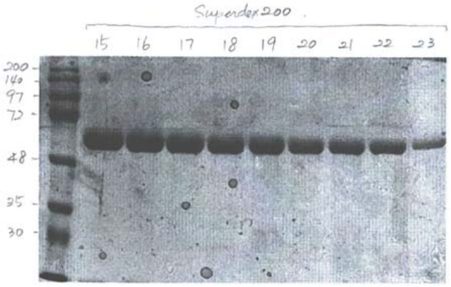

NusG was a gift of Prof. Jin Young Kang at Department of Chemistry, KAIST, Korea. NusG was expressed with a C-terminal His_6 tag from a cloned plasmid pRM1160 in *E. coli* BL21(DE3) and purified by using HiTrap IMAC, HiTrap Q, and HiLoad Superdex 75 columns, as recently described in *Cell* (2018) 173, 1650–1662. The final column elution and gel separation are shown below. This gel picture was previously shown in the supplementary data for the cited *Cell* (2018) paper.

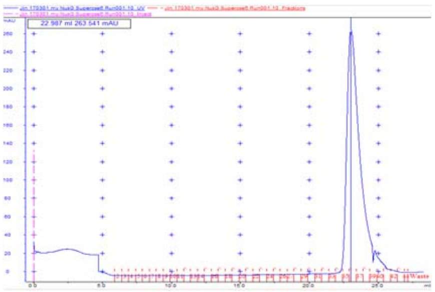

Marker sizes from the top:
250, 150, 100, 75 (purple),
50, 37, 25 (purple),
20, 15, 10 kDa

3. The choice of the experimental design which is rather speculatively based on "appearance" and "disappearance" of variously labeled RNA signals seems particularly ill-suited for the stated goal of elucidating the temporal order of elongation complex dissociation at intrinsic terminators. Six years had passed since publication of the report by Green and colleagues (Ref. 21), describing direct tracking of initiation and elongation RNA polymerases complexes along template by means of single-molecule fluorescence microscopy. Given the fact that, if needed, every component of the complex can be fluorescently labeled (core, sigma factor, and lacking from this work NusA, NusG, GreA, etc.), the fate of transcription complexes before, during, and after termination can be evaluated directly and transparently. Instead the authors chose an indirect method, which appears to be a significant step backwards compared to the single-molecule tracking of elongation complexes.

[Our response] We labeled nucleic acids rather than proteins in order to monitor dissociations of RNA and DNA from holoenzyme or core enzyme and also to examine structural changes of nucleic

acids. The last sentence of the Discussion section is rephrased (page 3, lines 62-64). Our experimental design was good enough to reveal the sequential order of labeled RNA and DNA dissociations. We examined some effects of σ, NusA and NusG on their dissociations (page 4, lines 115-121) but future studies are warranted to examine the other transcription factors. We agree that labeling of proteins would be sensible for examining dissociation of the factors from RNAP.

4. The effects of macromolecular crowding on cellular processes have been assumed to be significant for quite some time now, but recently a rather detailed and convincing experimental investigation into these effects (as they pertain to E. coli transcription) have been published (Chung et al. The effect of macromolecular crowding on single-round transcription by Escherichia coli RNA polymerase. Nucleic Acids Res. 2019 Feb 20;47(3):1440-1450. doi: 10.1093/nar/gky1277). In addition to the quite dramatic effects on transcription efficiency, macromolecular crowding mimicking the conditions within the cell would have significantly affected diffusion-limited processes.

[Our response] For this revision, we additionally repeated the experiments with the main template L+15 under a crowding condition with 15% PEG 8000, which turns out to substantially decrease PIFE occurrence to 63%. With this short DNA, the diffusion effect would be minimal. These results are newly described in the Results section (page 7, lines 206-207).

Technical issues:
1. The report is based on the commercially sourced E. coli RNA polymerase core and holoenzymes. The manufacturer (NEB) makes no claims regarding the exact composition or specific activities of these preparations, other than the ability to initiate transcription with or without additional supplementation with sigma factor, and the lack of nuclease activities. Common contaminants of RNA polymerase preparations (transcription factors, RNA and DNA helicases, etc.) will drastically affect the properties of the transcription complex, and, being usually present in sub-stoichiometric amounts, will introduce unknowable error/bias into the data. These considerations are the main reason for the extensive purification of the enzymes for in vitro experiments in general, and for the single-molecule ones in particular. Furthermore, if core and holoenzyme are to be compared in the same set of experiments, holoenzyme should be assembled using the same core and individually purified sigma subunit, as opposed to being procured independently and thus subject to additional variation between the preparations.

[Our response] Following the suggestion, we additionally repeated the main template experiments using new lab-purified *E. coli* RNA polymerase holoenzyme, and obtained the essentially same results (PIFE occurrence of 95 ± 6%) as those obtained using the holoenzyme purchased from New England Biolabs (91 ± 5%). The results are described in the Results section (page 5, lines 120-123), and these lab preparations are added to the Methods section (page 11, lines 314-324).

The lab-purified holoenzyme, another gift of Prof. Jin Young Kang, was reconstituted with the lab-purified core RNA polymerase and sigma-70 (RpoD) preparations that are described below. The core subunits were expressed from pVS11 encoding the alpha, beta, beta' and omega subunits and pACYCDuet-1_Ec_rpoZ encoding the omega subunit in *E. coli* BL21(DE3). They were extensively purified by using HiTrap IMAC affinity chromatography, polymin P precipitation, ammonium sulfate precipitation, Bio-Rex 70 ion exchange chromatography, and HiLoad Superdex 200 gel filtration chromatography, as previously described in *Protein Sci.* (2011) 20, 986-995, except for omission of a protease cleavage step. The final column elution and gel separation are shown below.

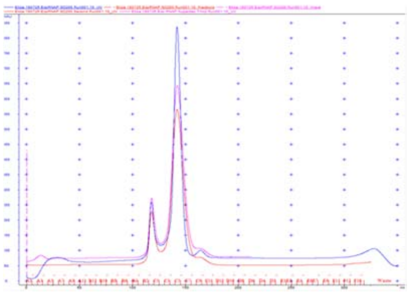

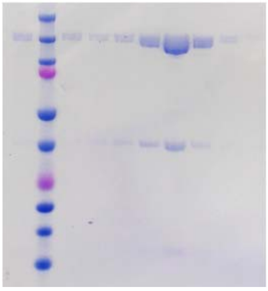

beta'
beta

alpha

omega

Marker sizes from the top:
250, 150, 100, 75 (purple),
50, 37, 25 (purple),
20, 15, 10 kDa

The RpoD (sigma-70) factor was expressed with a Hig_6-sumo tag from a cloned plasmid pETsumo in *E. coli* BL21(DE3) and then purified by using HiTrap IMAC HP, HiTrap Heparin HP, and HiLoad Superdex 200 columns as recently described in *Molecular Cell* (2017) 68, 388–397. The final column elution and gel separation are shown below.

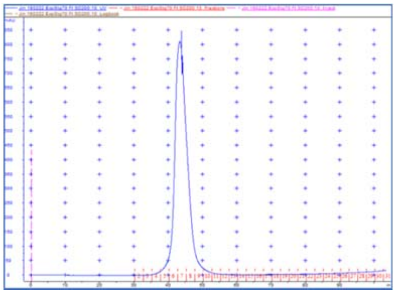

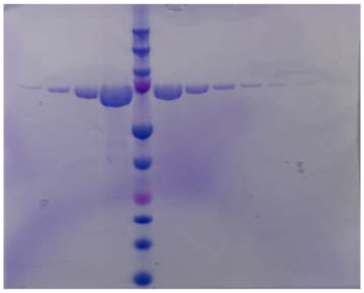

Marker sizes from the top:
250, 150, 100, 75 (purple),
50, 37, 25 (purple),
20, 15, 10 kDa

2. The provenance of the EcoRIQ111 is not described at all, which makes impossible to interpret the data concerning its "roadblocking" effect (depending on the expression and purification protocol, this protein may contain variable amounts of activity, DNA and/or associated proteins).

[Our response] The catalytically inactive EcoRI mutant E111Q was expressed with an N-terminal $flag_3$-tag and a C-terminal intein tag from a cloned plasmid in *E. coli* BL21(DE3), and the mutant without the intein tag was purified by using chitin-resin column chromatography and intein cleavage, as recently described in the supplementary data for *Nucleic Acids Res.* (2019) 47, 8337-8347. A description of this preparation is added to the revision in the Methods section (page 12, lines 331-336). This mutant preparation has been shown to bind its cognate sequence very tightly (K_d ≈ fM) and has been used as a protein obstacle on DNA in several previous experiments including those described in *PNAS* (2017) 114, E6322-E6331; *Mol. Cell* (2014) 54, 832-843 and *PNAS* (2007) 104, 12709-12713.

3. Purification of RpoD (sigma70) for fluorescent labeling is described in enough detail to conclude that a simple combination of Ni-affinity and heparin-affinity steps is not sufficient for the purposes of this work. Thus purified, RpoD preparations are contaminated with holoenzyme, and various sigma-binding proteins. Furthermore, this inadequately purified sigma was simply mixed with core to obtain holoenzyme in 50-fold excess. No attempt was made to purify the assembled holoenzyme (by combination of size-exclusion and ion-exchange chromatography); this in turn made unknowable the fraction of assembled holoenzyme, and created a great disparity between the commercial holoenzyme preparation (containing approximately 1 molecule of RpoD per complex) and in house assembled holoenzyme, which contained 50 times more. 50-fold excess of free sigma subunit present in sigma-dissociation experiments would drastically affect the sigma retention compared to the cellular conditions, where sigma factors were found to exist in sub-stoichiometric (relative total RNA polymerase).

[Our response] Unlike in bulk experiments, in single-molecule experiments, all free excess proteins are washed away before fluorescence monitoring, and only active transcription complexes on immobilized Cy5-DNA with Cy3-RNA are selected for data acquisition. Accordingly, excess sigma in the premix have been washed away to avoid such artefacts.

The concerned contaminations in the commercially sourced protein preparations were extensively removed in the lab preparations that are described above in our response to the technical issue #1. Especially, the lab-prepared sigma-70 was purified by not only Ni-affinity and heparin-affinity but also size-exclusion (as described on page 11, lines 322-324), and no contaminants were shown in the the final column elution profile and gel separation of elution fractions shown above.

4. When the authors examined the directionality of post-termination RNA polymerase 1-D diffusion, they employed DNA template L+112R attached to the cover slip via its downstream part as opposed to the upstream (non-transcribed) part in all other cases they studied. How such would this affect Cy5 PIFE fluorescence if RNA polymerase not only reached the end of the template but also the cover slip?

[Our response] Cy5 for PIFE and biotin for fixing are attached to the opposite ends in all our DNA templates. As shown in Figure 2A, the template L+112R has Cy5 (red dot) in the upstream end and biotin (black dot) in the downstream end, unlike all the other templates with upstream biotin and downstream Cy5. When RNA polymerase reaches the downstream end of L+112R and the cover slip, Cy5 on the opposite end would not be touched by the polymerase. In order to minimize misunderstanding, a phrase is added on line 153, page 6. On this template, Cy5 PIFE would occur only when RNA polymerase approaches the upstream end away from the cover slip through either non-transcriptional backward diffusion or transcription on the opposite strand.

5. In the case of the double promoter setup for re-initiation probing, the authors did not explain how they ruled out a possibility that the second RNA polymerase occupied the downstream promoter and that they observed initiation from the already occupied promoter when all nucleotides were present.

[Our response] When a control experiment for reinitiation was performed with a negative control template that lacks the first, upstream transcription unit, the initial stalling complexes on the remaining transcription unit were very few in the initial transcription buffer that lacks UTP, probably because the starting sequence is 5'-UGC. This is now described in the Results section of the revision (page 9, lines 238-241).

Minor:
The treatment of transcription termination in the introduction makes a significant omission of the key mechanistic paper (PMID: 18158897). This paper explains the universal molecular pathway leading to intrinsic termination, which occurs via conformation changes of RNAP and does not involve any forward translocation or RNA shearing proposed by Larson et al. (ref. 9). Another obvious omission is the paper describing the original observation of persistent sigma70 retention during elongation (PMID: 11525730). That paper also demonstrated facilitated recycling by such a subpopulation of sigma70-retaining elongation complexes. Both papers must be properly cited and discussed in the revised manuscript.

[Our response] These two mentioned papers are cited on page 3, line 59 and on page 5, line 132, and added to the References section as #10 and #13. Thanks for all the comments and suggestions.

Response to the Comments of Reviewer #2:

The authors present experimental evidence for the maintenance of an RNA polymerase/DNA complex subsequent to RNA transcript release during termination. Using a variety of template lengths and the occurrence of fluorescence enhancements, they show that this complex likely undergoes 1D diffusion on the DNA in the absence of any RNA. Finally, they show that this diffusing complex is capable or re-initiation on a downstream promoter. These observations run counter to transcriptional dogma for bacteria, and as such, will be of interest to a wide range of researchers. While potentially significant, there are significant concerns that should be addressed prior to recommending publication.

(1) I wonder if 38nt is a well defined EC. It would be reassuring to see the same behavior with a longer transcriptional unit.

[Our response] Following the suggestion, additional PIFE experiments were performed with a new long DNA template T257/L+15 that contains a 257-bp transcription unit in place of 38-bp unit of L+15. The PIFE occurrence is 86 ± 2% on T257/L+15, similar to 91 ± 5% on L+15. These results are described on page 5, lines 124-127 in the Results section.

(2) Salt! The experiments are being performed at 20 mM NaCl which is quite low compared to typical in vitro transcription experiments (~150 mM) and to estimates of in vivo conditions. This is of critical importance as the maintenance of the polymerase/DNA interaction should be quite salt dependent. Here I am specifically referencing both the concentration of salt, but also the identity (i.e. NaCl vs. KGlu). Far beyond reporting the conditions, an actual titration of salt concentration should most certainly be performed. At what concentration does retention of RNAP after termination become disfavored and how does this concentration compare to estimates of in vivo salt concentrations?

[Our response]

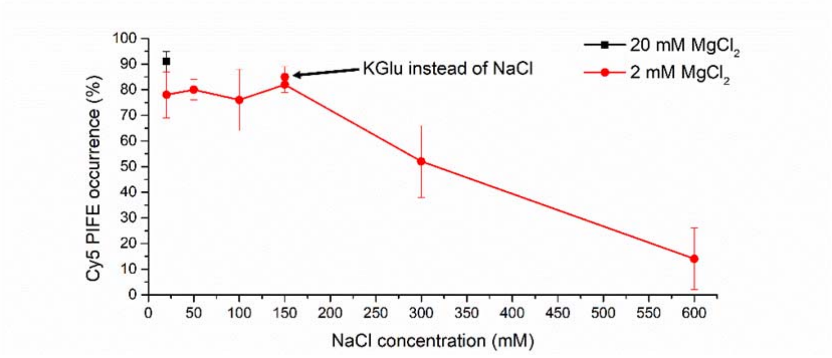

For this revision, we additionally repeated the main template experiments at varying NaCl concentrations (from 20 to 600 mM) but at fixed 2 mM $MgCl_2$, which is closer to physiological conditions than the previous 20 mM $MgCl_2$. The [NaCl] variation from 20 to 150 mM little affects Cy5 PIFE occurrence; 78 ± 9% at 20 mM, 80 ± 4% at 50 mM, 76 ± 12% at 100 mM, and 82 ± 3% at 150 mM. However, at non-physiological 300 and 600 mM NaCl, the PIFE occurrence decreases much to 52 ± 14% and 14 ± 12%, respectively. When KGlu replaces NaCl at 150 mM, PIFE occurrence is still 85 ± 4%, indicating that KGlu versus NaCl makes little difference. On the other hand, [$MgCl_2$] reduction from 20 to 2 mM (at 20 mM NaCl) decreases the PIFE occurrence from 91 ± 5% to 78 ± 9%. These salt variation effects are newly described in the Results section (page 7, lines 196-207), and their data are newly shown in the Supplementary Fig. 8.

(3) Fig. 1E, what is the jump from 0.5 to 1 in the red laser excitation? This figure would benefit greatly from a listing of what each (D,E, and G) are referring to. Some kind of subtitles for example (D: readthrough, E: termination) Also some quantification of how often each behavior is observed would also be helpful in the

figure itself (as described in the text).

[Our response] The mentioned jump is Cy5 PIFE. Figures 1D, 1E, and 1G are renumbered as Figures 2a, 2b, and 3, respectively, and modified to have new wordings such as "readthrough", "termination", "PIFE", and percentages, as suggested.

(4) Line 122: Under no cases did sigma dissociate before the RNA? Did the authors mean that IF sigma was observed in the stalled elongation complex, it remained until after RNA dissociation? Or did they mean that they ALWAYS observed sigma in complexes where they observed RNA? This needs be specified. (I imagine it is the former as even the studies showing retention of sigma show ~30% dissociation upon entering the elongation complex I believe).

[Our response] Yes, we observed no cases where sigma dissociates before RNA, as already stated on page 5, line 138. Yes, we mean that the sigma retained in the stalled complex mostly remains on DNA after RNA release. A phrase is added to the pertinent sentence (page 5, lines 137-138).

(5) Type 2 under reinitiating section. No (re)-initiation, but what is the evidence for dissociation of RNAP? It seems rather than although no secondary RNA is produced, the labeling strategy makes it ambiguous as to whether RNAP is still present.

[Our response] The title of type 2 is changed from "dissociation" to "no reinitiation" on Fig. 5b and on page 8, lines 225-226, because the observed failure of reinitiation can be caused by not only dissociation of RNA polymerase from DNA but also inactivation of DNA-bound polymerase such as sigma dissociation and aberrant conformation change, although the RNA polymerase dissociation may be more frequent than its inactivation. This renaming does not affect our conclusions at all.

(6) Also, in the case of 1D diffusion, doesn't one also expect to see cases of re-initiation on Promoter 1? This could be seen by the re-occurrence of a green signal. If this is not observed normally, certainly in the presence of the EcoR1 barrier, the model predicts it?

[Our response] In order to examine possibility of such feedback-type reinitiation on the original transcription unit by post-terminational RNA polymerase, we performed additional experiments with a newly constructed DNA template that encodes transcripts containing the probing sequence (five 21-nt repeats) that can be probed by the previously used Cy5-labeled DNA oligomer. This probing sequence is located in the first, upstream transcription unit in this new template, unlike the previous template that has it in the second, downstream unit. We observed a positive probing signal, indicating that post-terminational RNA polymerase can diffuse back to the original promoter for reinitiation in a feedback fashion. These descriptions are added to the revision in the Results section (page 9, lines 247-252), and the data are shown in new Fig. 6. The Discussion section is accordingly revised on page 10, lines 282-287. Please note that the elongation-resuming buffer (NTP injection buffer) does not contain Cy3-ApU in all the experiments, so its green signal is not expected from such feedback-type reinitiation.

(7) line 226: what is the evidence that this mechanism is "dramatically" faster compared to de novo transcription initiation? Won't this depend on the free concentration of RNAP and the distance between terminators and promoters?

[Our response] Now removed is the word 'dramatically', and added is a sentence "This effect would be more prominent when promoter-terminator distance is shorter and RNAP concentration is lower." on page 10, lines 276-278.

(8) I don't understand how (re)-initiation would contribute to transcriptional bursts as it would have to diffuse backwards against the stream of other RNAPs. Unless the authors mean that the polymerase would diffuse within a loop to re-encounter the first promoter, but I don't understand how a loop would interact with 1D diffusing RNAPs.

[Our response] Forward diffusion towards downstream would contribute to RNA polymerase accumulation in a downstream promoter. Backward diffusion necessary for accumulation in an upstream promoter would be more feasible under less crowding conditions. This is discussed on page 10, lines 295-297.

In case of promoter-terminator loops, like those observed in some eukaryotic genes, upstream accumulation would be more feasible by inter-segment transfer across the loop than sliding or hopping. The pertinent sentence is now rephrased (page 11, line 306). Thanks for all the comments and suggestions.

Additionally, Supplementary Tables 1, 2 and 3 and Supplementary Figures are now supplemented with the numbers of analyzed molecules (*n*) in replicated experiments with each DNA template.

REVIEWERS' COMMENTS:

Reviewer #1 (Remarks to the Author):

The authors demonstrated a good-faith effort to address the bulk of criticisms.
The authors included PEG800 in the experiments and, although this addition does not fully replicate macromolecular crowding in the cell, it should sensitize the reader to the inherent complexities of the mechano-chemical processes such as transcription.

The authors also repeated experiments with a lab-purified preparation of RNAP, which allowed for greater transparency and reproducibility of the experimental findings.

In response to my criticism the authors also prepared and analyzed the effects of a subset of elongation factors, namely NusA and NusG; this, albeit, partial expansion of the transcription factor repertoire, serves as welcome and informative reminder of the differences between the transcription process in vivo and in vitro.

The same can be said about the expansion of the template length - still not entirely native, but a substantial improvement over the originally submitted one.

The authors also provided a more nuanced and measured description of their findings and their relation to the complexities of transcription in its cellular context. Altogether the manuscript has been substantially improved.

I found a couple of minor issues that need to be fixed:

lines 58,59: [9] paper cannot be contradictory to [10] as the two papers use completely different approaches. The authors should revise this line to something like this: "… although biochemical studies demonstrated an allosteric mechanism of termination, which does not involve RNA shearing or RNAP forward translocation [10]".

lines: 304-308: This part of the discussion is too speculative and should be removed to avoid confusion, as the authors' results with a roadblock appear to contradict this looping model.

Reviewer #2 (Remarks to the Author):

I thank the authors for their careful responses to the first round of review. They have done a great deal of new experiments that greatly enhance the manuscript and make it acceptable for publication.

I have only a couple of remaining points and suggestions regarding figure 1B, the description of how/when free polymerases are washed out of the experiment (to prevent de novo binding) and using the EcoRI block to prove that secondary initiation is truly generated via diffusion. See below.

(1) This result is convincing.

(2) This is a nice addition to the manuscript.

(3) OK. I think the figure in 1B is misleading because you are really thinking of termination only as release of RNA. If, as the figure shows, the polymerase also dissociates then you wouldn't expect to see any PIFE. I recommend carefully describing in the text and in the figure what you mean by "termination". In fact, to be precise, perhaps using "transcript release" instead of termination may prevent misunderstandings.

(4) OK. Thanks for the clarification. Surprising!

(5) OK.

(6) A point of general clarification please. In the description of the assay (in general), the authors do not describe a wash step to remove free polymerases from solution prior to initiation with NTPs. Presumably, this is something that is done to prevent the de novo binding of a soluble polymerase, but I can't find it in the paper. Are the unbound complexes flown out during the flow in of NTPs? Much of the results point to the interpretation of a single polymerase bound to the DNA anyway (distance dependences of PIFE, EcoR1 block, lack of loss of fluorescent sigma during diffusion), but a comment here of exactly how the experiment was done would be recommended.

Also, as the template for re-initiation experiments has a EcoRI binding site, presumably the authors looked at whether the block prevents downstream initiation on the secondary promoter, but I do not see that experiment described. Has/can it been done? In the absence of a wash step, it would be a nice control.

(7) I would also ask what is the evidence that this process can "accelerate" promoter search. Again, I would guess that this depends on the polymerase concentration. Evidence that 1D diffusion can lead to re-initiation events is really what is shown.

(8) OK.

Author Response to the Comments of Reviewer #1:

The authors demonstrated a good-faith effort to address the bulk of criticisms. The authors included PEG800 in the experiments and, although this addition does not fully replicate macromolecular crowding in the cell, it should sensitize the reader to the inherent complexities of the mechano-chemical processes such as transcription. The authors also repeated experiments with a lab-purified preparation of RNAP, which allowed for greater transparency and reproducibility of the experimental findings. In response to my criticism the authors also prepared and analyzed the effects of a subset of elongation factors, namely NusA and NusG; this, albeit, partial expansion of the transcription factor repertoire, serves as welcome and informative reminder of the differences between the transcription process in vivo and in vitro. The same can be said about the expansion of the template length - still not entirely native, but a substantial improvement over the originally submitted one. The authors also provided a more nuanced and measured description of their findings and their relation to the complexities of transcription in its cellular context. Altogether the manuscript has been substantially improved.

I found a couple of minor issues that need to be fixed: lines 58,59: [9] paper cannot be contradictory to [10] as the two papers use completely different approaches. The authors should revise this line to something like this: "… although biochemical studies demonstrated an allosteric mechanism of termination, which does not involve RNA shearing or RNAP forward translocation [10]".
[Our response] The phrase is revised to "... termination mechanisms with RNAP forward hypertranslocation and RNA shearing [9], which are not involved in an allosteric mechanism demonstrated by biochemical studies [10]."

lines: 304-308: This part of the discussion is too speculative and should be removed to avoid confusion, as the authors' results with a roadblock appear to contradict this looping model.
[Our response] This paragraph is entirely deleted, as suggested.

Author Response to the Comments of Reviewer #2:

I thank the authors for their careful responses to the first round of review. They have done a great deal of new experiments that greatly enhance the manuscript and make it acceptable for publication.
I have only a couple of remaining points and suggestions regarding figure 1B, the description of how/when free polymerases are washed out of the experiment (to prevent de novo binding) and using the EcoRI block to prove that secondary initiation is truly generated via diffusion. See below.

(1) This result is convincing.
(2) This is a nice addition to the manuscript.
(3) OK. I think the figure in 1B is misleading because you are really thinking of termination only as release of RNA. If, as the figure shows, the polymerase also dissociates then you wouldn't expect to see any PIFE. I recommend carefully describing in the text and in the figure what you mean by "termination". In fact, to be precise, perhaps using "transcript release" instead of termination may prevent misunderstandings.
[Our response] The Fig. 1b scheme did not include our major finding of termination with RNA release but RNAP retention, so was indeed misleading. Fig. 1b is now changed to show the three different consequence diagrams that are named "Readthrough," "Termination of sequential dissociation," and "Termination of concurrent dissociation." The fourth theoretically possible consequence can be another termination event with RNAP dissociation but RNA retention on DNA, but is omitted here because it has never been observed and is not expected to be likely either. This comment enlightens me to revise Fig. 2 also to cover all three different events. Its previous version did not include the data for termination of concurrent dissociation. Therefore, new Fig. 2c is

added being entitled as "Termination of concurrent dissociation (3%)."
The title of Fig. 2b "Termination (33%)" could be misleading although PIFE (91%) was shown, and is revised to "Termination of sequential dissociation (30%)" without saying that PIFE is 91%.
Now the Fig. 1b scheme would match well with the Fig. 2 data.
Termination was already defined as RNA release in an early part of the Results section, and is now on line 107 of page 4 in the change-tracked revision file. As recommended, however, distinction between Fig. 2b and 2c events is made clear in the text (page 5) and in the Fig. 2 legend.

(4) OK. Thanks for the clarification. Surprising!
(5) OK.
(6) A point of general clarification please. In the description of the assay (in general), the authors do not describe a wash step to remove free polymerases from solution prior to initiation with NTPs. Presumably, this is something that is done to prevent the de novo binding of a soluble polymerase, but I can't find it in the paper. Are the unbound complexes flown out during the flow in of NTPs? Much of the results point to the interpretation of a single polymerase bound to the DNA anyway (distance dependences of PIFE, EcoR1 block, lack of loss of fluorescent sigma during diffusion), but a comment here of exactly how the experiment was done would be recommended.
[Our response] An extensive washing step was carried out as a routine between the immobilization step and the elongation resumption step in all our experiments. A phrase regarding the washing is added to the Results section at the end of the "Construction of fluorescent transcription complex" subsection (page 4) and to the Methods section at the end of "Single-molecule transcription termination experiments" subsection (page 13).

Also, as the template for re-initiation experiments has a EcoRI binding site, presumably the authors looked at whether the block prevents downstream initiation on the secondary promoter, but I do not see that experiment described. Has/can it been done? In the absence of a wash step, it would be a nice control.
[Our response] The reinitiation blocking experiment had been done and was already described in the last subsection of the Results section. The sentence could have been overlooked because it was very brief and split between pages 8 and 9 in the previous manuscript. It is now on line 258, page 9 in the change-tracked revision file, but extended with "... (Supplementary Table 3), supporting that reinitiation is generated by 1D diffusion." Additionally, the next sentence on σ supplementation experiment is also extended with an interpretation.

(7) I would also ask what is the evidence that this process can "accelerate" promoter search. Again, I would guess that this depends on the polymerase concentration. Evidence that 1D diffusion can lead to re-initiation events is really what is shown.
[Our response] The sentence is now revised to "This study provides the first direct evidence that post-terminational RNAP's 1D diffusion on DNA is frequent and long enough to efficiently facilitate reinitiation."

(8) OK.

Thanks for constructive suggestions and enlightenments.
Additionally, some measurements are revised because more replicate experiments were performed in order to reduce standard deviations.
